# *Operando* optical fiber monitoring of nanoscale and fast temperature changes during photo-electrocatalytic reactions

**DOI:** 10.1038/s41377-022-00914-5

**Published:** 2022-07-13

**Authors:** Zhi Li, Yongguang Xiao, Fu Liu, Xiangyu Yan, Daotong You, Kaiwei Li, Lixi Zeng, Mingshan Zhu, Gaozhi Xiao, Jacques Albert, Tuan Guo

**Affiliations:** 1 Guangdong Key Laboratory of Environmental Pollution and Health, School of Environment, Jinan University, Guangzhou, 511443 China; 2 Guangdong Key Laboratory of Optical Fiber Sensing and Communications, Institute of Photonics Technology, Jinan University, Guangzhou, Guangdong 511443 China; 3 Department of Electronics, Carleton University, Ottawa, K1S 5B6 Canada; 4 Advanced Electronics and Photonics Research Center, National Research Council of Canada, Ottawa, K1A 0R6 Canada; 5 Southern Marine Science and Engineering Guangdong Laboratory (Zhuhai), Zhuhai, 519000 China

**Keywords:** Imaging and sensing, Fibre optics and optical communications

## Abstract

In situ and continuous monitoring of thermal effects is essential for understanding photo-induced catalytic processes at catalyst’s surfaces. However, existing techniques are largely unable to capture the rapidly changing temperatures occurring in sub-μm layers at liquid-solid interfaces exposed to light. To address this, a sensing system based on a gold-coated conventional single-mode optical fiber with a tilted fiber Bragg grating inscribed in the fiber core is proposed and demonstrated. The spectral transmission from these devices is made up of a dense comb of narrowband resonances that can differentiate between localized temperatures rapid changes at the catalyst’s surface and those of the environment. By using the gold coating of the fiber as an electrode in an electrochemical reactor and exposing it to light, thermal effects in photo-induced catalysis at the interface can be decoded with a temperature resolution of 0.1 °C and a temporal resolution of 0.1 sec, without perturbing the catalytic operation that is measured simultaneously. As a demonstration, stable and reproducible correlations between the light-to-heat conversion and catalytic activities over time were measured for two different catalysis processes (linear and nonlinear). These kinds of sensing applications are ideally suited to the fundamental qualities of optical fiber sensors, such as their compact size, flexible shape, and remote measurement capability, thereby opening the way for various thermal monitoring in hard-to-reach spaces and rapid catalytic reaction processes.

## Abstract (web summary)

*Operando* monitoring of highly localized and rapid temperature changes during photo-involved catalysis reactions at liquid-solid interfaces via narrow bandwidth fiber-optic spectral combs.

## Graphical abstract

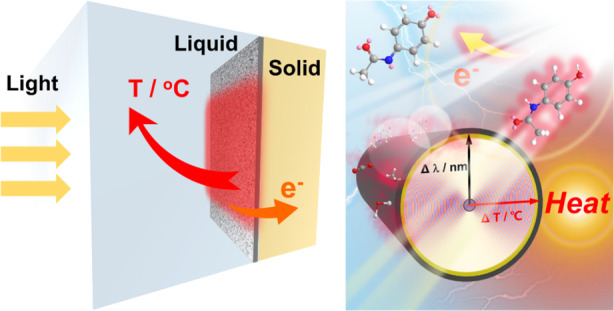

## Introduction

Solar-to-chemical energy conversion through photocatalysis or photo-electrocatalysis is one of the most attractive solutions to address the current energy crisis and global warming^1–3^. In these kinds of photo-involved catalytic reactions, photothermal effects are always generated at the surface of the catalyst. Such thermal effects in turn drive the catalyst’s microscopic reactions and influence the quantitative structure-activity relationships of catalyst^4^. To further understand the correlation between thermal effects and photo-involved catalytic processes, it is urgently needed to continuously monitor the local temperature in situ, i.e., at the catalyst surface, from the macroscopic scale down to the microscopic scale^5^. The common methods for monitoring the surface temperature of catalysts are based on contact methods (e.g., thermocouple, scanning thermal microscopy) and non-contact methods (e.g., infrared thermal imager)^6–9^. However, such methods mainly focus on the macroscopic scale and most of them are only fit for catalytic reactions at gas-solid interfaces.

**Fig. 1:**
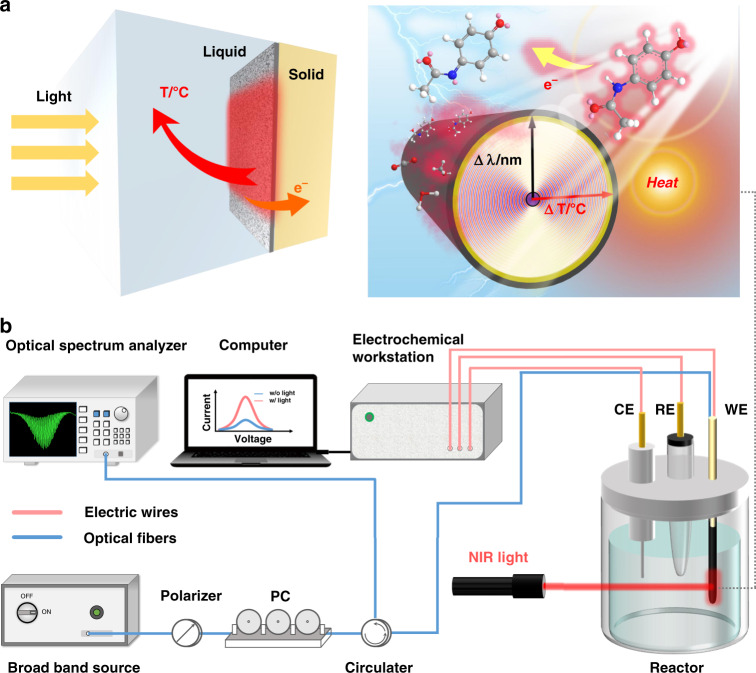
Concept of operando monitoring thermal effects in photo-involved catalytic reactions via an optical fiber. **a** Photothermal effect in photo-involved catalytic reactions. ΔT is the photo-induced temperature increase at catalyst interface and Δ λ is the wavelength shift of cladding mode resonance. **b** Optical fiber sensing principle and system for *operando* monitoring light-to-heat at liquid-solid catalyst interface

It has long been a challenge to measure temperatures at the microscale, particularly at liquid-solid interfaces, due to the fact that heat quickly diffuses into the surrounding solvent medium, thus creating a yet unsolved problem for studying catalytic processes in operation (Fig. 1a). Recent reports mainly rely on advanced theoretical models and sophisticated laboratory tools like fluorescent or luminescence thermometry^5,10–17^, surface-enhanced Raman spectroscopy^18–20^, and similar techniques. Unfortunately, such techniques are still unable to capture the surface-localized and rapidly changing temperature near the catalyst’s surface in operando, with high accuracy, speed, and fine spatial resolution. Moreover, another unaddressed issue is how to remove the crosstalk between environmental temperature fluctuations so as to precisely measure the heat “definitely” generated at the catalytic interface. For this to occur, fundamentally new techniques must be developed for implanted sensors that are compatible with the harsh and hard-to-reach catalytic space.

Fiber-optic sensors (FOSs) provide a promising approach to these challenges, as they are made of chemically resistant silica glass benefiting from high sensitivity, instantaneous response, and electrical immunity^21,22^. Among them, fiber Bragg gratings (FBGs) have been widely investigated for temperature, strain, pressure, and vibration measurement, by monitoring the wavelength shift of the Bragg core mode in reflection^23,24^. However, the critical chemical parameters such as refractive index (RI) are still missing. More recently, tilted fiber Bragg gratings (TFBGs) were proposed, and the internal tilt favors the coupling of light to modes guided by the cladding instead of the core. Since the cladding diameter is much larger (almost 100 times the wavelength), tens to hundreds of cladding modes could be excited at a specific wavelength, resulting in a fine spectral comb with desirable refractive index region from 1.0 to 1.45^25,26^. This enables the development of a high-performance sensor for (bio-) chemical sensing^27–29^, electroactive biofilms^30^, and supercapacitors^31^. Recently, progress reported by Tarascon et al managed to decode the refractive index of electrolyte, thermal, and stress distribution of anode (cathode) inside commercial Na(Li)-ion cells by structured FBG and TFBG in an operando way^32,33^. All preliminary findings above convince the potential of optical fiber sensors for optimization of thermal measurement and chemical reactions of batteries for better performance. From this starting point, researchers are looking for more advanced tools which can measure physical, chemical, and electrochemical parameters simultaneously with higher temporal and spatial resolution.

Here, in contrast with previous efforts in this area, we use a commercial single-mode fiber coated with carbon nanotubes (CNT) which absorb near-infrared light to generate heat at the surface of an electrode made up of a nanoscale gold (Au) layer also deposited on the fiber cladding. In addition to acting as a system electrode, the Au layer enables the TFBG to measure the fast and highly localized temperature changes occurring at the CNT-gold boundary when light impinges on it. The fast simultaneous in situ measurement of both surface temperature and electrical performance allows for more advanced diagnostics catalyst–reactant interfaces, without perturbing the catalysis operation **(**Fig. 1b**)**. By monitoring the spectral wavelengths of the superfine optical comb of cladding mode resonances excited by the TFBG in the fiber core, the local surface temperature can be measured and distinguished from the system temperature. When connected to an electrochemical workstation, the Au film present on the optical fiber conducts the electrical signals generated by the photo-involved electrocatalytic reactions located at the same position. Therefore, we can simultaneously obtain both the catalytic performances and photothermal effects via a tiny optical fiber sensor, and decode their correlation unambiguously. Apart from the surface probing by the cladding mode resonance combs, the proposed TFBG also provides a resonance for light guided in the fiber core (hereafter named the “Bragg” resonance) to measure the bulk surrounding temperature at the same time and separately, thereby enabling the measurement of the near-surface temperature changes associated with the photo-electrocatalytic reactions unambiguously. As a demonstration to prove the feasibility of the proposed method, measurements were performed during two representative electrochemical processes driven by near-infrared (NIR) continuous wave (CW) laser irradiation: electro-oxidation and electro-detection. Stable and reproducible correlations between the real-time light-to-heat and catalytic activities have been found, which helps to provide a fundamental understanding of photo-involved catalytic processes and mechanisms. This simple-to-implement method fills an important gap in the current catalytic process and heat monitoring in real-time and it offers a scalable solution for screening sub-μm-scale thermal events adjacent to the catalytic interface with high accuracy and speed. It can also be easily integrated as part of existing catalytic components for *operando* monitoring.

## Results

### Generation of photothermal effects on a coated optical fiber

As discussed above, in situ monitoring interfacial temperature in the proximity of catalyst surfaces is still a challenge. We try to address this long-lasting question using a gold-coated fiber-optic sensor (Au-FOS). Figure 1b and S1 show the all-fiber-coupled electrochemical fiber-optic sensing system including a broadband light source, polarizer, polarization controller, circulator, Au-FOS (immersing in the reactor), and optical spectrum analyzer. An electrochemical workstation, performing electrochemical measurements, is used as a reference to the optical signal extracted from Au-FOS.

**Fig. 2:**
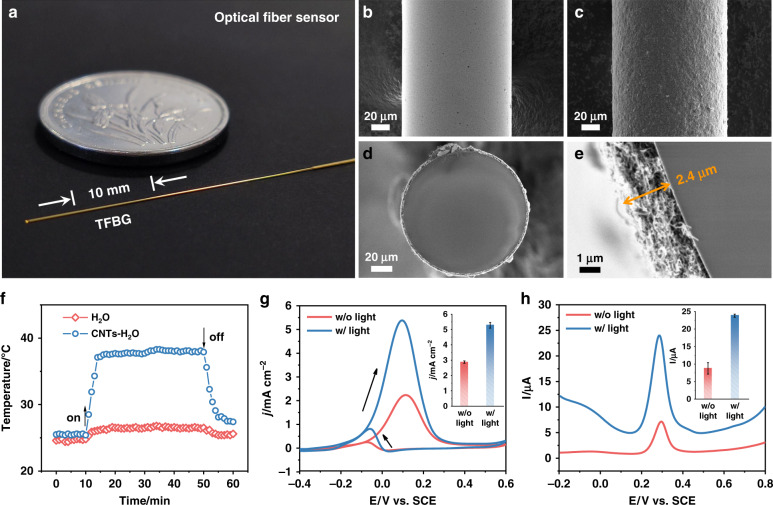
Photothermal effects in photo-electrocatalytic reactions over optical fiber surface. **a** Photograph of Au-FOS. **b**–**e** SEM images of Au-FOS (**b**), CNTs/Au-FOS (**c**) and its cross-sections (**d**, **e**). **f** Temperature responses of CNTs-H_2_O vs. H_2_O with the light on and off. **g**, **h** Comparisons of CNTs/Au-FOS responses w/ and w/o light irradiation: **g**, CV behaviors on sensor in 1 M ethanol and 1 M KOH solution. **h** Differential pulse voltammetry (DPV) behaviors of 20 µM APAP on sensor in 0.1 M phosphate buffer (pH = 7.0). The insets in **g** and **h** show the corresponding histograms of electrocatalytic activities. Error bars result from the standard deviation from five measurements

A special design of the fiber sensor is at the end of the fiber. It is coated by Au film and used as a mirror to provide near 100% reflection. The mirror also highly enhances the attenuation of cladding mode due to the fact that the propagation modes go through the grating twice. Compared to the transmission by fixing both ends of grating, the reflection configuration eliminates the effect of strain-induced inside the chemical reactor such as thermal expansion. The entire Au-FOS is compact (125 µm in diameter and 10 mm in length), as shown in Fig. 2a. Meanwhile, a scanning electron microscope (SEM) image shows the surface of the Au-FOS and of a representative electrocatalysis layer of carbon nanotubes (CNTs) over the Au film (Figs. 2b, c). The thickness of coated CNTs is about 2.4 µm (Figs. 2d, e). The detailed characterizations of the CNTs, including fine morphology, X-ray diffraction (XRD) pattern, and optical properties are shown in Figs. S2–S4. The photothermal conversion ability of CNTs was recorded by using an infrared imager. Figure 2f and S5 show that the temperature of the reactor vessel water in the presence of CNTs increased from 25.4 to 38.1 °C within 4 min of NIR light illumination by a laser beam incident from outside, confirming the ability of CNTs to convert light to heat^34^.

By using CNT coatings as the electrocatalysts, two model electrochemical reactions, including electrochemical ethanol oxidation and detection of acetaminophen (APAP) under light illumination, were chosen to probe the influence of photothermal effects during photo-involved electrocatalytic processes. Before photo-electrocatalytic reactions, the optimum electrochemical conditions of CNTs coated Au-FOS (CNTs/Au-FOS) were evaluated by electrochemical impedance spectroscopy (EIS), cyclic voltammetry (CV), electrochemically active surface area (ECSA), pH, accumulation potential and time in the Figs. S6–S8. As shown in Fig. 2g, h, both electrocatalytic activities of ethanol oxidation and APAP detection for CNTs/Au-FOS with (w/) light illumination are much higher than those without (w/o) light illumination. For comparison, only slight changes in electrochemical signals were observed for pure Au-FOS w/ and w/o light illumination (Fig. S9). These results suggest that the enhanced activities in CNTs/Au-FOS upon light illumination are caused by the photothermal effects from the coated CNTs during light irradiation. To explore further the photothermal effect from light illumination, cooling circulating water was used to diffuse out the generated heat. Figure S10 shows that the electrocatalytic activities were decreased compared with the ambient conditions but still higher than those w/o light illumination. This phenomenon indicates that the cooling circulating water can only maintain the macroscopic temperature of the reaction vessel but not fully that of a microscopic layer near the catalyst surface. To fully correlate photo-electrochemical reactions to the photothermal effect, it is needed to monitor the local temperature at the microscopic scale of the catalyst surface in real-time during the photo-electrocatalytic reaction.

### *Operando* monitoring of thermal effects at the liquid-solid catalyst interface

**Fig. 3:**
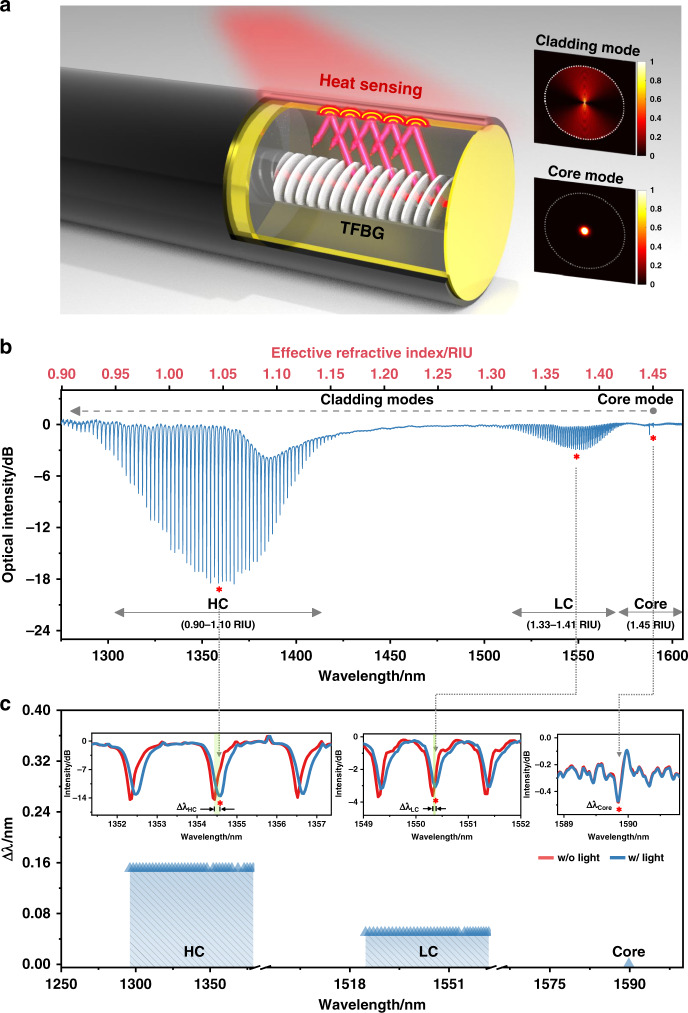
Principle and demonstration of operando monitoring thermal effects via a gold-coated TFBG optical fiber sensor. **a** Schematic illustration of a CNTs/Au-FOS with a TFBG in the fiber core and an Au mirror (4 μm) at the end of fiber. The insets are the numerical simulation of guided mode field intensity distributions for cladding mode and core mode. **b** Reflection spectrum of the fiber sensor in water, and the corresponding wavelength of high-order cladding (HC) modes for HC (90–108) and low-order cladding (LC) mode for LC (39–61), and core. **c** Wavelength shifts (∆λ) and corresponding spectral responses (the insets: HC (99), LC (49) and core) due to photo-induced catalytic processes over the Au film. (Video S1 for HC mode response and Video S2 for core mode response are provided)

Contrary to the case for standard fiber Bragg gratings, the planes of the refractive index modulation of TFBGs were written with a predefined tilt relative to the longitudinal axis of the fiber, as shown in Fig. 3a. Such tilted grating fringes provide an additional resonant mechanism which produces a high-density spectral comb of narrowband resonances at near-infrared wavelengths (with a spectral width of the resonance ~0.1 nm), suitable for high accuracy temperature measurements. Here, an infrared imager was first used to verify the capability of TFBGs to detect surface temperatures. Figures S11 and S12 show that the infrared thermal imager indicates the same temperature as the TFBG at the gas-solid surface of fiber in air, but that it cannot image (and get the fiber surface temperature) after passing through the electrolyte liquid. More importantly, compared to other optical detection methods based on ultraviolet-visible light, the near-infrared light used in the TFBG-assisted fiber-optic sensor probes the metal-electrolyte surface temperature from the opposite side of the metal layer, and its longer wavelength allows for deeper penetration in the metal and thus greater sensitivity to temperature changes. Figure 3a shows the structure of the TFBG and the intensity mode profiles of the core mode and of a representative cladding mode, showing why the cladding mode can probe the surface while the core mode cannot. Temperature changes in fiber gratings are measured via spectral comb shifts of representative resonances. While cladding mode resonances shift in response to both surface and environmental temperature changes, the core mode can only sense the environmental one.

The tilt of the grating is an important parameter that determines which set of cladding modes is exciting. Here, a 37° angle is chosen to maximize coupling to cladding modes that are closer to their guiding cutoff, and therefore with maximum sensitivity due to larger penetration in the metal and higher mode dispersion. Figure 3b shows the transmission spectrum of the 37° TFBG (corresponding to an internal tilt angle of 23°, due to refraction of the interference fringes of the light pattern used to fabricate the grating at the glass-air boundary). The core mode effective index of 1.45 produces resonance with the longest wavelength near 1590 nm and hundreds of additional resonances from 1250 nm to 1570 nm are due to cladding modes. These cladding modes appear in two main subsets: one group located at wavelengths ranging from 1480 nm to 1530 nm is marked as “LC”: they are EH/TM and HE/TE lower-order cladding modes (radial order numbers of 39–61) with effective indices from 1.33 to 1.41. Another group of cladding modes that have resonances at wavelengths ranging from 1250 nm to 1375 nm is marked as “HC”, i.e., higher-order modes (radial order numbers of 90–108) with effective indices between 0.90 and 1.10. These “HC” are the most sensitive to the surface temperature of the gold film. Finally, a special design aspect of the proposed sensor is that the optical fiber is coated with a 300~500 nm thickness of Au. Since the real part of the refractive index of Au at 1300 nm wavelength is about 0.31^35^, which gives an index contrast of almost 1.0 with the cladding glass, such thickness of Au ensures that the evanescent fields of the cladding modes penetrate deeply into the metal but not fully across it. Therefore, the mode fields can only probe the metal layer and remain insensitive to the surrounding medium (Fig. S13). Further confirmation is provided by the fact that the temperature sensitivity of HC modes of a gold-coated TFBG with an additional CNT layer ceases to change when the Au thickness reaches the 300–500 nm range (Fig. S14).

Figure 3c shows typical wavelength shift responses (∆λ) of each group of cladding modes during photo-induced catalytic processes at the gold film surface. The 150 pm wavelength shift of HC modes is nearly 3 times stronger than that of LC modes (since their mode fields penetrate deeper into the gold), while the core mode experiences a negligible wavelength shift (within measurement uncertainty) during the same catalytic process. This further shows that a standard FBG temperature sensor, which measures only core mode resonance shifts, cannot measure these localized temperature responses. It should be noted that, due to light impinging on the CNT-gold boundary from only one direction, this will induce non-uniform heating over the whole fiber surface and therefore slightly deform the spectra of each cladding resonance (see the red and blue spectral curves of the insets in Fig. 3c, the cladding resonances of HC and LC modes become broadened in bandwidth and weakened in amplitude). This problem can be effectively solved by using a spectral fitting method (e.g., polynomial curve fitting). In the following sections, we will carefully explain a scalable solution that uses wavelength shifts of the spectral comb of resonances to rapidly and accurately quantify the local temperature at the interface of the µm-scale CNT-gold layers where the photo-electrocatalysis occurs.

### Quantifying surface thermal effects at the liquid-solid catalyst interface with high spatial and temporal resolutions

**Fig. 4:**
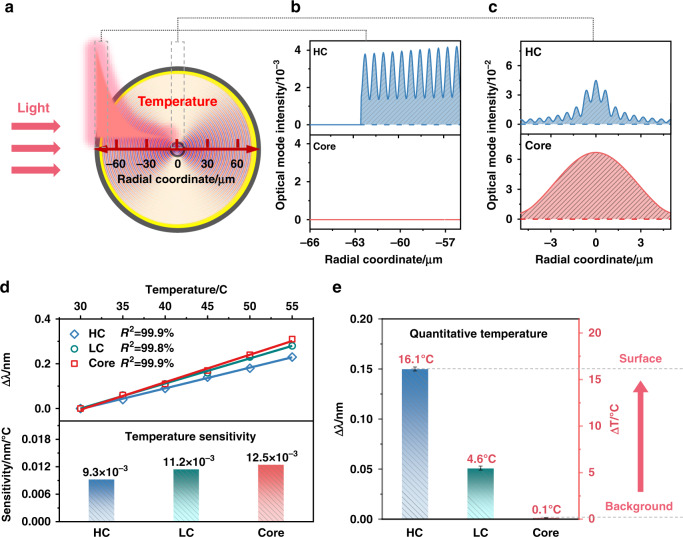
Operando thermal measurement methodology at the liquid-solid catalyst interface with high spatial and thermal resolutions. **a** The numerical simulation of mode field distributions in the fiber cross-section (excited by a TFBG) for light-to-heat measurements and approximate radial temperature distribution. **b**, **c** Mode power distributions near the fiber surface and within the 8.2 μm diameter fiber core. **d** Thermal sensitivity of the HC (99) mode and of the core mode to ambient temperature changes. **e** Temperature increases at the fiber surface determined from the wavelength shift of the HC (99), LC (49), and core mode with 1 min of CW laser irradiation, according to the sensitivity obtained from **d**, and simultaneous observation of no wavelength shift of the core mode resonance. Error bars result from the standard deviation from five measurements

As shown schematically in Fig. 4a, when NIR light is absorbed by the CNT film on the Au-FOS surface, heat is generated locally, thus enabling electrocatalysis. Due to the very high thermal conductivity and the thickness of this CNT film (2.4 µm), compared to the 62.5 µm radius of the optical fiber, heat generated at the surface is dissipated faster along the fiber surface and outward in the electrolyte liquid than towards the fiber center because of the relatively lower thermal conductivity of glass. A temperature gradient thus forms from the fiber surface towards the center (as shown by the red shading in Fig. 4a). Furthermore, the cladding mode fields extend over the whole cross-section of the fiber and penetrate the Au coating while the core mode fields are constrained within a 10 µm diameter cross-section at the center of the fiber (Figs. 4b, c show simulated power distributions for the HC (99) and core mode). The calibration of the thermal sensitivities of the HC mode (9.3 pm/°C), LC mode (11.2 pm/°C), and core mode (12.5 pm/°C) to global temperature change were obtained with the whole fiber heated up to stable temperatures in an oven (Fig. 4d**)**, which is used in Fig. 4e to calibrate the surface temperatures by the wavelength shifts observed during the catalytic processes from Fig. 3c. The fact that the core mode shifts very slightly in Fig. 4d (within a measurement uncertainty of 0.1 °C) validates the mechanism proposed in Fig. 4a, i.e., fast and highly localized temperature changes occurring at the CNT-gold boundary when light impinges on it (a temperature increase of 16.1 °C quantified by the wavelength shift of HC mode), while the center temperature of the optical fiber remains unchanged which maintains thermal equilibrium with the liquid electrolyte. Any slight shift of the core mode resonance would reflect a global change in the reactor temperature and should be subtracted from the HC mode shift so as to reveal the heat “definitely” generated at the catalytic interface. Here in our experiments, the wavelength interrogation accuracy is 1 pm (using an optical spectrum analyzer from Yokogawa), yielding a temperature accuracy of the order of 0.1 °C.

**Fig. 5:**
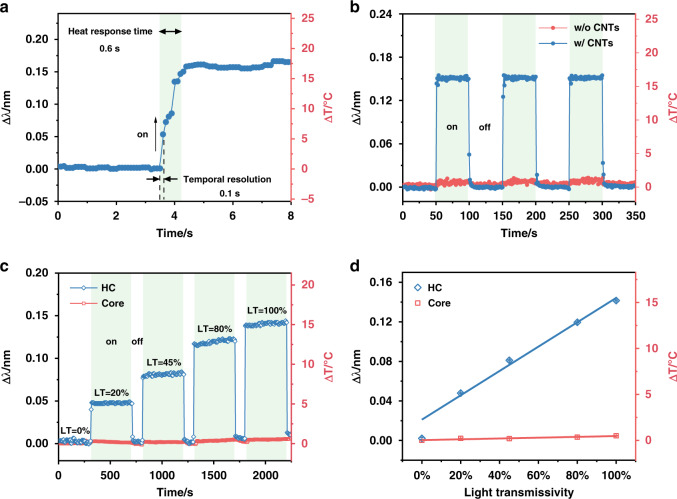
Quantification of the temporal response of photothermal effects at the liquid-solid catalyst interface. **a**, **b** Wavelength shifts of typical resonances w/ and w/o CNT coating before and after light switch on, showing the response time of light irradiation (**a**) and three cycles with light on/off (**b**). **c** Real-time wavelength shifts response of HC (99) mode and core mode with increasing light power levels, controlled by filters with transmissivities from 0 to 100%. **d** The linear relationships between the temperature changes for HC and core modes and the light power level

Moreover, the optical spectrum data acquisition speeds with current instruments are sufficient to measure wavelength shift data over limited spectral widths on a 0.1 s time scale. Fig, 5a shows that the temperature increases and stabilizes in about half a second following the start of the irradiation of the CNT layer with the near-infrared light source. Furthermore, it was verified that the temperature increase is directly caused by the presence of the CNT layer (and not by direct heating of the Au layer by the light), as shown in Fig. 5b. And it also shows a reproducible wavelength shift with no hysteresis or baseline drift over three on/off light cycles over a period of 6 min. Finally, Figs. 5c, d provide evidence of a direct, linear relationship between the near-infrared light intensity and the wavelength shift (hence on the surface temperature) by inserting various light attenuators with transmissivity levels of 0%, 20%, 45%, 80%, and 100%. Regardless of the irradiating light power, no temperature increase is observed at the fiber core (Figs. S15 and S16).

### Correlating photothermal effects and catalytic activities

**Fig. 6:**
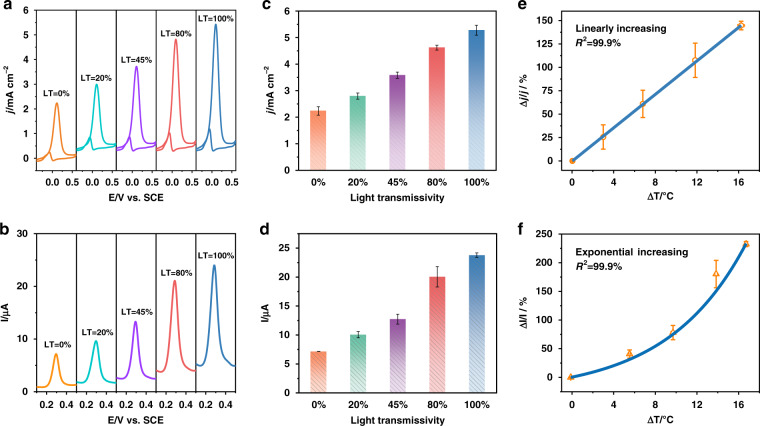
Decoding the relationship between light power, surface temperature, and photo-electrocatalytic activities. **a**, **b** CV changes in 1 M ethanol and 1 M KOH solution (**a**) and DPV changes of 20 µM APAP in 0.1 M phosphate buffer (pH = 7.0) (**b**) with increasing light transmissivity (LT). **c**, **d** The corresponding histograms of electrochemical signals *vs*. power level for ethanol oxidation (**c**) and APAP detection (**d**). **e**, **f** Relationship between the electrocatalytic activity of ethanol oxidation (**e**), APAP detection (**f**), and the catalyst surface temperature. Error bars result from the standard deviation from five measurements

In the catalytic process, the temperature is one of the most basic parameters influencing the catalytic activity in addition to catalyst structure and composition^4^. The kinetics of the surface catalytic process is usually interpreted in terms of surface- and diffusion-controlled processes, in which the corresponding surface-controlled process is highly dependent on reaction rate and temperature^36–39^. To decode the relationships of light-to-heat generated temperature and related catalytic activity in the photo-involved catalytic process, two typical electrocatalytic processes were investigated under different light irradiation levels: ethanol oxidation and detection of APAP. First, it was determined that the electrocatalytic processes could be detected with the CNT-Au fiber coating acting as the working electrode. In the absence of light irradiation, all three mode groups have negligible wavelength shifts and the electrical measurements proceed as expected (Fig. S17), indicating that the FOS does not perturb the catalytic operation. Then, the changes in the electrochemical signals of the CV curves of 1 M ethanol and the DPV curves of 20 µM APAP under various light power irradiation levels are shown in Figs. 6a, b, respectively. The corresponding histograms for the average and standard deviation in each case are shown in Figs. 6c, d. It can be seen that the oxidation currents of ethanol and APAP gradually increase, as the transmissivity of the optical attenuator increases. These trends are similar to those observed under global temperature changes imposed on the system by immersion in a water bath (Fig. S18), thus confirming that the increased activity is due to photothermal effects at the electrode surface. The photo-induced increased electrolytic activity was then plotted as a function of the simultaneous surface temperature measurements from the HC mode shifts and fitted for the two processes in Figs. 6e, f. A linear equation is obtained for ethanol oxidation while a nonlinear equation is fitted for the electro-detection of APAP by using CNTs as electrocatalyst. The fit parameters are added in Supplementary Information (Table S1).

These different trends arise from the kinds of reactions involved (shown in Fig. S19). An irreversible reaction occurs in the process of ethanol oxidation while the target molecules of APAP follow a reversible reaction at the surfaces used in electro-detection. Furthermore, Figure S20 shows the effect of different scan rates on the ethanol oxidation and APAP detection processes. It can be concluded that the electrochemical reaction of ethanol electro-oxidation at the surface of CNTs is a diffusion-controlled process^40^ while APAP detection is a surface-controlled process^41^. Another reaction mode of photo-electrocatalytic of H_2_O_2_ detection is added, and a similar phenomenon is observed where the performance of electrocatalyst was exponential increase with the increase of light transmissivity (Fig. S21), owning to H_2_O_2_ detection is a surface-controlled process (Fig. S22)^42^. Upon light irradiation, the CNTs convert light to heat, immediately raising the local temperature and accelerating the activation of target molecules involved in the electrochemical reactions. What is shown here is that differences between the accelerating surface-controlled process and the linearly increasing diffusion-controlled process can be revealed in operando by the simultaneous local temperature measurement provided by the TFBG. Therefore, our method provides an easy-to-implement method for in situ and continuous monitoring of the correlations between surface temperature and catalytic kinetics in the real-time photo-involved catalytic reactions.

## Discussion

An in situ and continuous monitoring of the surface temperature on a sub-μm-scale near a liquid-solid catalyst interface is provided by using a TFBG imprinted in a commercial single-mode fiber and coated with a nanoscale Au film acting as a working electrode. Unlike previous works on FBG- and TFBG-based fiber sensing in electrochemistry^30–33^, the fiber here is not used to measure electrical signals or electrolyte charge density through detection of surface plasmon effects, or to simply act as system thermometers in the electrolyte surrounding the electrodes. The CNT/gold coating allows for separate measurements of global reactor temperature (from shifts of the core guided mode) and of the metal surface, thanks to high-order cladding modes with high sensitivity to the metal temperature, without perturbation of the photo-electrocatalytic reactions. A thermal resolution of 0.1 °C, a fast temporal resolution of 0.1 s is demonstrated by a light-to-heat-to-surface temperature change at a liquid-solid catalyst interface. These features are absent from the “FBG part” of the sensor response, indicating that conventional fiber grating thermometers are unable to provide the same information. Moreover, a stable and reproducible correlation between light-to-heat and catalytic activities has been measured in real-time for two photo-activated electrochemical processes, electro-oxidation of ethanol and electro-detection of APAP. Surface temperature measurements revealed a diffusion-controlled process of electro-oxidation (linear dependence on surface temperature) and a surface-controlled process for electro-detection (nonlinear relationship of electrocatalytic activity with light-to-heat generated temperature). This fundamental understanding thus gained is an example of the added benefits provided by using metal-coated TFBG FOS in operando for electrochemical sensing systems research and development and eventually in deployed catalytic power plants.

While the interrogation tools used herein include expensive instrumentation incompatible with widespread use in monitoring power plants with a large number of photo-catalytic electrodes, work is underway to develop customized, multiplexed laser diode-based interrogation systems compatible with mass production techniques (Fig. S23). Finally, accurate multi-point temperature measurements and mapping the temperature distribution are a prerequisite for a correct interpretation of catalysis results of light-powered chemical reactions obtained with plasmonic catalysts^43^. These new tools will help to improve the efficiency, reliability, and cost-effectiveness of systems and processes and thus help to advance the progress in the use of solar power as a viable energy source.

## Materials and methods

The material and instruments involved in this work are discussed in the Supplementary Information (Text S1 and S2).

### Fabrication of tilted fiber gratings

The 23-degree (10-mm length) TFBGs were fabricated in boron- germanium co-doped highly photosensitive single-mode fiber (FIBERCORE PS1250/1500) using a small-frame excimer laser (Bragg Star Industrial, Coherent, Inc.) emitting 193 nm pulses with 3 mJ per pulse at a frequency of 200 Hz. The laser beam spot passed through an 1110 nm period uniform phase mask to produce the permanent periodic refractive index in the fiber core, and was scanned along the fiber axis at a speed of 20 μm/s. The tilt angle of the grating was obtained by rotating the fiber and phase mask around an axis perpendicular to the plane of the fiber axis and laser beam by 37 degrees (corresponding to an internal angle in the fiber of 23 degrees relative to the fiber axis, due to refraction of the light at the fiber interface). This angle ensures that numerous cladding mode resonances at wavelengths ranging from 1250 nm to 1550 nm wavelength are excited (Fig. S24).

### Surface functionalization

A 300~500 nm thick Au film was deposited on the surface of TFBG probe using an RF magnetron sputtering system (Polaron Instruments TRI-S500 metal coating system operated at a pressure of 5 Pa in Argon gas with a discharge current of 0.2 A and a voltage of 0.4 kV). To achieve a smooth Au coating on the fiber surface, a 2~3 nm thick chromium layer was first coated as a binder between the silica fiber surface and the gold film. Meanwhile, the uniformity of the film around the fiber surface was ensured by rotating the fiber along its axis at a constant speed of 0.5 rad/s inside the vacuum chamber. Afterward, the grating probe was further coated with CNTs by solution-dipping in a CNTs (70.0 wt%) dimethylformamide dispersion for 1 min. Finally, the sensor was dried in an oven at 60 °C for 5 min.

### Sensing system

The all-fiber-coupled fiber-optic sensing system employed is shown in Fig. 1b and S1. The optical reflection spectra were measured with a broadband light source (Golight, OS-EB-LD-1450-400-30-S-FA) and detected by an optical spectrum analyzer with a nominal wavelength accuracy of 1 pm (Yokogawa, AQ6380). A polarizer and polarization controller (PC) (both from Shanghai Fsphotonics Technology Ltd.) was used to linearize the input light polarization parallel (P-polarized) or perpendicular (S-polarized) to the grating tilt direction, after which the reflected light from the fiber grating sensor probes was extracted from the feed fiber by a circulator for routing to the optical spectrum analyzer. An electrochemical workstation (CH Instruments, CHI760E) is used for performing the conventional photo-electrochemical measurements carried out in parallel and simultaneously with the optical measurement of the fiber sensing probe.

### Electrochemical measurements

All electrochemical measurements were performed with an electrochemical workstation (CH Instruments, CHI760E). A three-electrode system was prepared with the metal-coated fiber-optic sensor, Pt wire, and saturated calomel electrode (SCE) as the working, counter, and reference electrodes, respectively. A 2 W 808 nm infrared laser (HX58081000D-AL, Tengxing Optoelectronics Technology Co., Ltd.) was used as NIR light source to photo-generated temperature increases at the catalytic surface. Different NIR power levels were obtained by inserting fixed optical transmission filters between the laser and the catalytic surface.

The electrocatalytic activity of ethanol oxidation was carried out by CV measurements, with a scan rate of 100 mV s^−1^ and a potential range from −0.4 to 0.6 V. The electrochemical behavior of APAP detection on the sensor was analyzed by DPV, with an applied potential range from −0.2 to 0.8 V, an incremental potential of 0.004 V, an amplitude of 0.05 V and a pulse period of 0.05 s. The whole detection process adopts a stripping mode, and the accumulation potential and time were set as −0.2 V and 150 s, respectively. The scan rate of the CV was set to 100 mV s^−1^, with the potential range of −0.2 to 0.6 V.

### Theory of temperature measurement using TFBG

For TFBGs, the wavelength shifts induced by temperature changes (∆*T*) across the whole fiber for the core and for the *r*^th^ cladding mode can be determined by Eqs. 1 and 2:^25^

1 $$\Delta \lambda _B = \left( {2\frac{{N_{eff}^{core}}}{{\cos \left( \theta \right)}}\frac{{d{\it{\Lambda }}}}{{dT}} \,+\, 2\frac{{\it{\Lambda }}}{{\cos \left( \theta \right)}}\frac{{dN_{eff}^{core}}}{{dT}}} \right)\Delta T$$

2 $$\Delta \lambda ^r = \left( {\frac{{\left( {N_{eff}^{core} + N_{eff}^r} \right)}}{{\cos \left( \theta \right)}}\frac{{d{\it{\Lambda }}}}{{dT}} \,+\, \frac{{\it{\Lambda }}}{{\cos \left( \theta \right)}}\left. {\frac{{d\left( {N_{eff}^{core} + N_{eff}^r} \right)}}{{dT}}} \right)} \right)\Delta T$$

where $N_{eff}^{core}$ and $N_{eff}^r$ are the effective refractive index of the core and *r*th cladding mode, respectively, *Λ* is the grating period, *θ* is the grating tilt angle. Considering uniform heating without any axial strain (since the sensor is fixed at one point only), the dominant term in each equation is the thermo-optic effect ($dN/dT$ is of the order of 10^–5^ for silica glass and this is 20 times larger than the thermal expansion coefficient of silica (0.5 × 10^–6^/°C)). Then the difference between the wavelength shifts of core and cladding modes is:

3 $$\Delta \lambda ^r - \Delta \lambda _B = \left( {\frac{{\it{\Lambda }}}{{\cos \left( \theta \right)}}\frac{{d\left( {N_{eff}^{core} - N_{eff}^r} \right)}}{{dT}}} \right)\Delta T$$

As a result, the cladding mode resonances “lag” behind the Bragg resonance (they have slightly lower sensitivities than the Bragg resonance) when the fiber is heated up, see Fig. 4d.

### Optical simulation

The guided mode inside the fiber in terms of transverse electric power density was simulated by using the FIMMWAVE software package (Photon Design Inc.) with an 8.2 μm core diameter and a refractive index of 1.449311, a 125 μm cladding diameter with a refractive index of 1.444078, a 300 nm thick-coated gold at 1300 nm wavelength with a refractive index of 0.3071 + 8.995i^35^. The outer layer of the simulation is a 20 μm thick layer of air (air refractive index of 1.00027).

## Supplementary information

Supplementary information

Movies S1 Video for HC mode response

Movies S2 Video for core mode response

## Data Availability

The data that support the plots within this paper and another finding of this study are available from the corresponding author upon reasonable request.
