# Supplementary material for: Operando optical fiber monitoring of nanoscale and fast temperature changes during photo-electrocatalytic reactions
Source: Light Sci Appl. 2022 Jul 13;11:220. doi: 10.1038/s41377-022-00914-5 (PMC9279429; doi:10.1038/s41377-022-00914-5)
Supplement: Supplementary file 1 — Supplementary information [file 41377_2022_914_MOESM1_ESM.docx]

**Supplementary Information**

***Operando* optical fiber monitoring of nanoscale and fast temperature changes during photo-electrocatalytic reactions**

Zhi Li^1,6^, Yongguang Xiao^2,6^, Fu Liu^3,6^, Xiangyu Yan^2^, Daotong You^2^, Kaiwei Li^2^, Lixi Zeng^1^, Mingshan Zhu^1,*^ Gaozhi Xiao^4,*^, Jacques Albert^3,*^ and Tuan Guo^2,5,*^

^1^ Guangdong Key Laboratory of Environmental Pollution and Health, School of Environment, Jinan University, Guangzhou, Guangdong

511443, China

^2^ Guangdong Key Laboratory of Optical Fiber Sensing and Communications,

Institute of Photonics Technology, Jinan University, Guangzhou, Guangdong

511443, China

^3^ Department of Electronics, Carleton University, 1125 Colonel By Drive, Otrawa K1S 5B6, Canada

^4^ Advanced Electronics and Photonics Research Center, National Research Council of Canada, Ottawa K1A 0R6, Canada.

^5^ Southern Marine Science and Engineering Guangdong Laboratory (Zhuhai), Zhuhai 519000, China.

^6^ These authors contributed equally to this work.

Correspondence and requests for materials should be addressed to:

M.Z. ([zhumingshan@jnu.edu.cn](mailto:zhumingshan@jnu.edu.cn))

G.X. (George.Xiao@nrc-cnrc.gc.ca)

J.A. ([Jacques_Albert@carleton.ca](mailto:Jacques_Albert@carleton.ca))

T.G. ([tuanguo@jnu.edu.cn](mailto:tuanguo@jnu.edu.cn))

**Supplementary Texts**

**Text S1.** **Materials.**

Dimethylformamide dispersion of CNTs (70.0 wt%) was purchased from Xianfeng Nano Material Technology Co., Ltd. in Nanjing, China. Acetaminophen (APAP) was purchased from Aladdin, Shanghai. Disodium hydrogen phosphate (Na_2_HPO_4_), sodium dihydrogen phosphate (NaH_2_PO_4_), ethanol, potassium hydroxide (KOH), potassium ferrocyanide (K_4_[Fe(CN)_6_]) and potassium ferricyanide (K_3_[Fe(CN)_6_]), were obtained from Sinopharm Chemicals Reagent Co., Ltd, China. 0.1 M phosphate buffer was prepared by mixing 1M Na_2_HPO_4_ and 1M NaH_2_PO_4_ solution, and the pH value was adjusted by adding HCl and NaOH. All compounds are of analytical grade and are commercially available. All chemicals were used without further purification.

**Text S2.** **Instruments.**

X-ray diffraction (XRD, D2 PHASER, Bruker, Germany) is used to investigate the crystalline structure of samples at 30 kV and 10 mA. The morphologies of all samples were tested by using a field-emission scanning electron microscope (SEM, Ultra-55, Germany) and transmission electron microscope (TEM, JEOL JEM-2100F, Japan). The optical properties of the sample were measured by (JASCO, V-770, Japan), and BaSO_4_ was used as the reference. The temperature is recorded by a Fluke TiS65 infrared imager (Fluke Corporation). Electrochemical impedance spectroscopy (EIS) was performed at an initial potential of 0.175 V, a range of 1.0-1.0×10^6^ Hz and amplitude of 5 mV. The EIS are tested in 2.5 mM K_3_[Fe(CN)_6_]/K_4_[Fe(CN)_6_] solution with 0.1 M potassium chloride (KCl).

**Supplementary Figures**

**
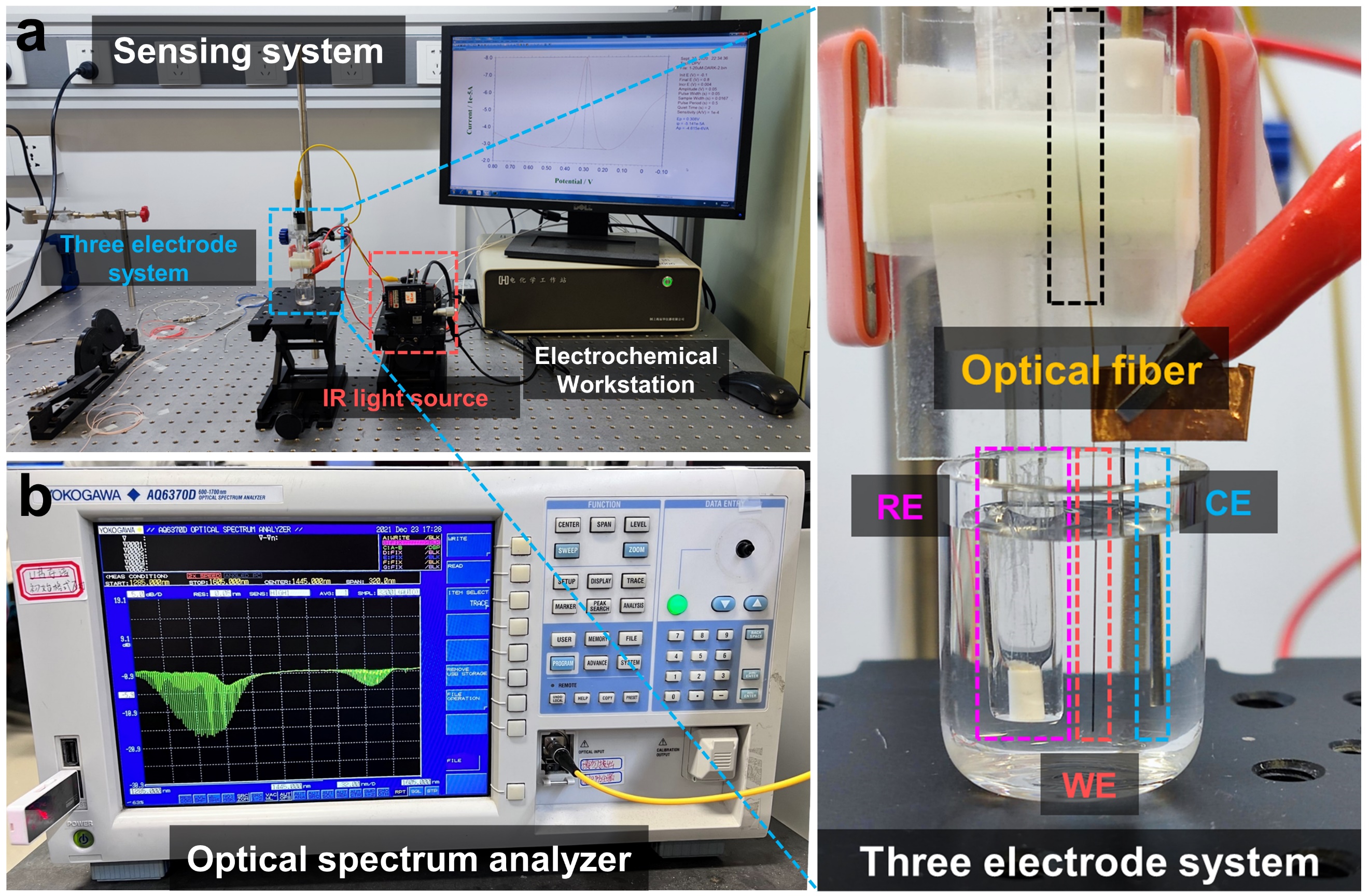
**

**Figure S1:** Photographs of the experimental set-ups. (a) Sensing system, (b) Optical spectrum analyzer used for optical signal detection and analysis.


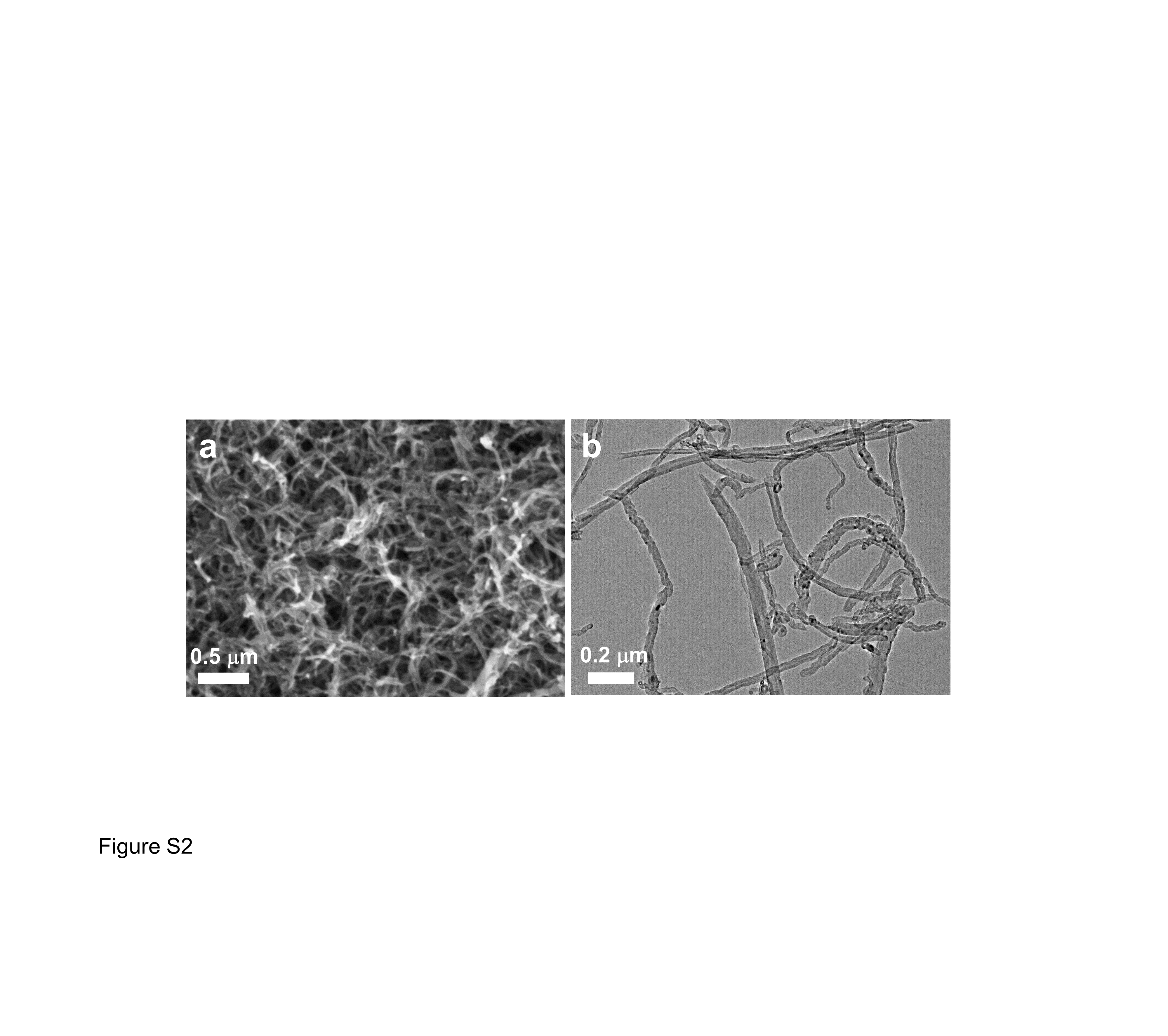


**Figure S2:** Morphology of CNTs. (a) SEM image, (b) TEM image.


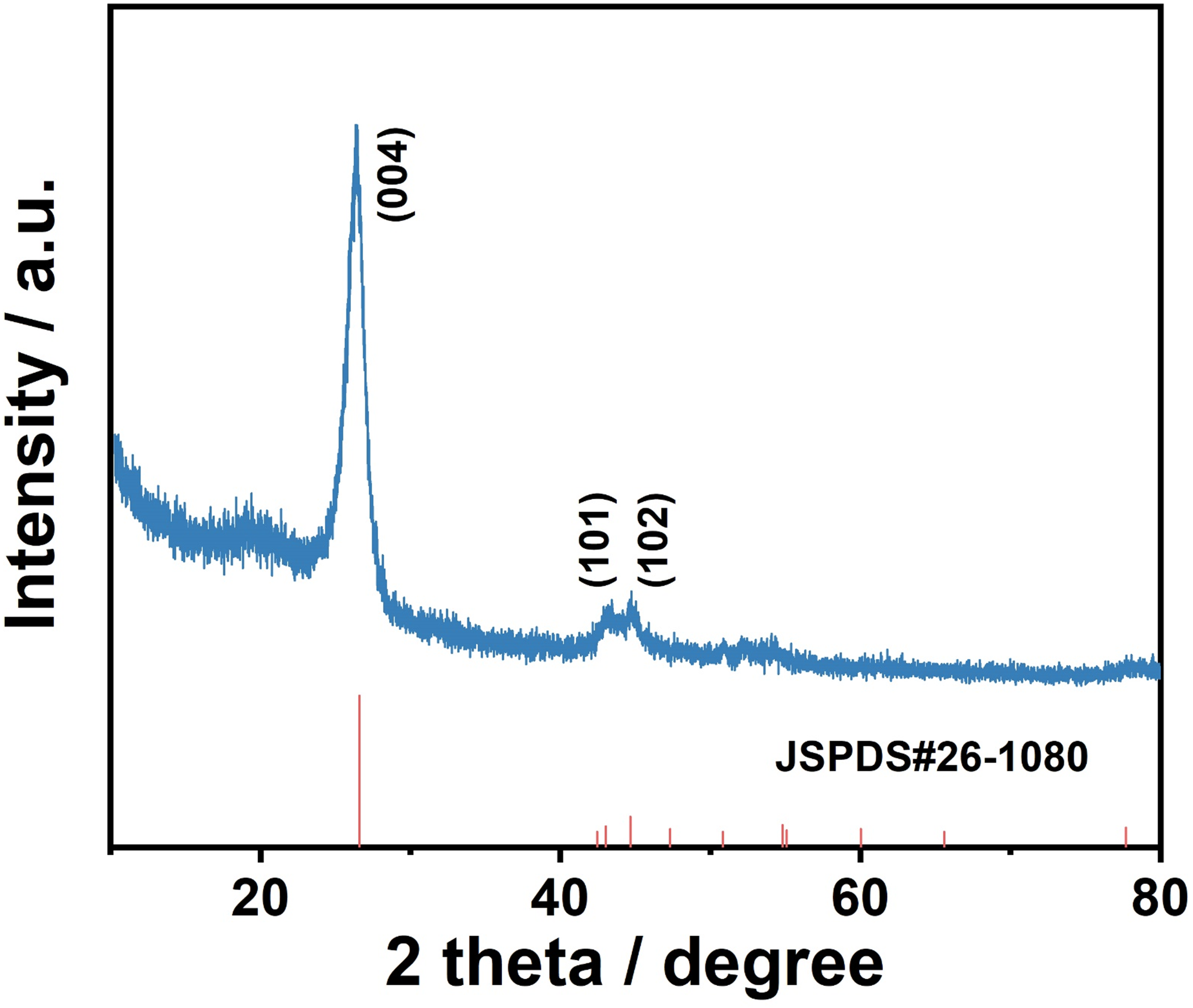


**Figure S3:** XRD pattern of CNTs.


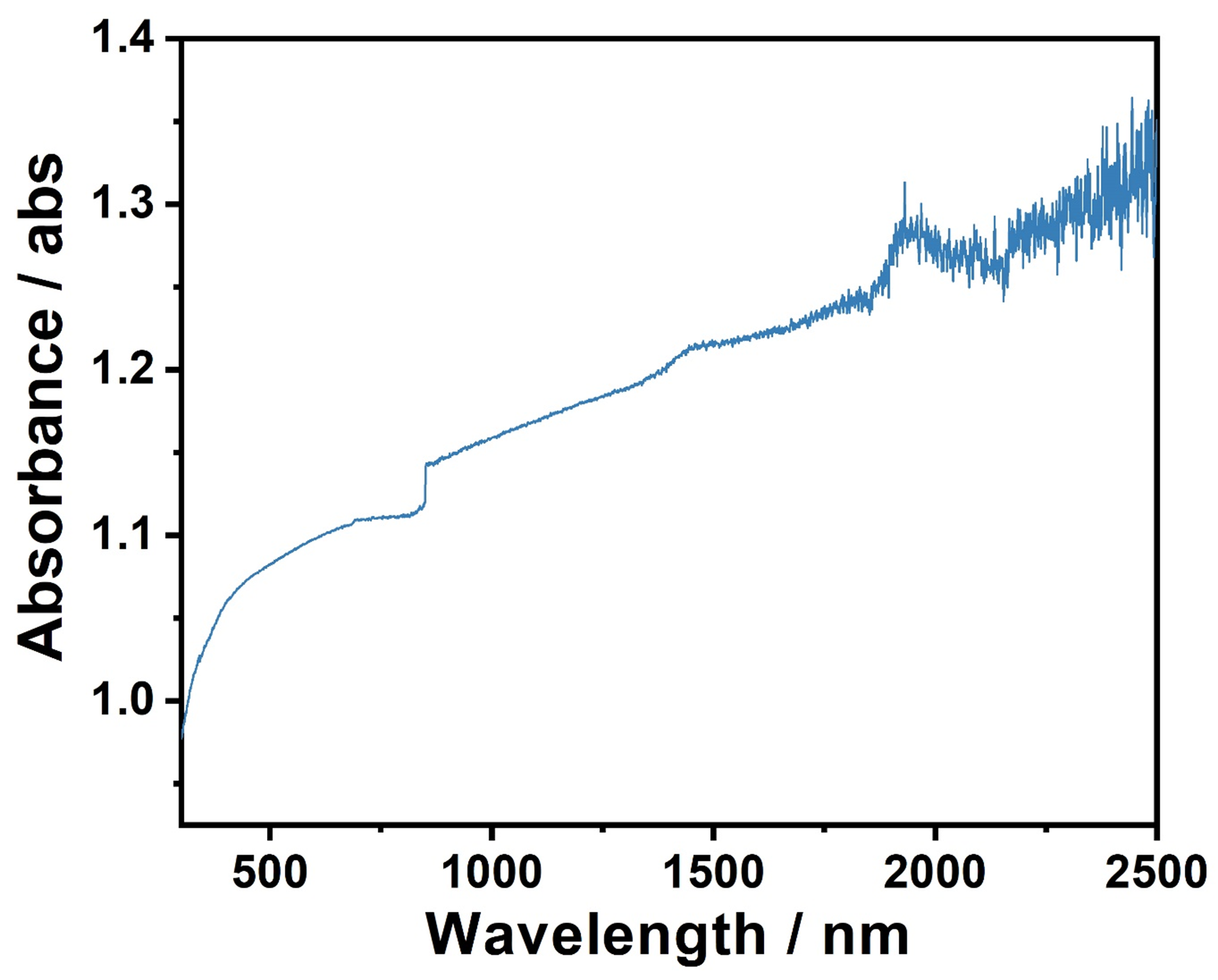


**Figure S4:** UV–Vis diffuse reflectance spectrum (UV-Vis DRS) of CNTs.


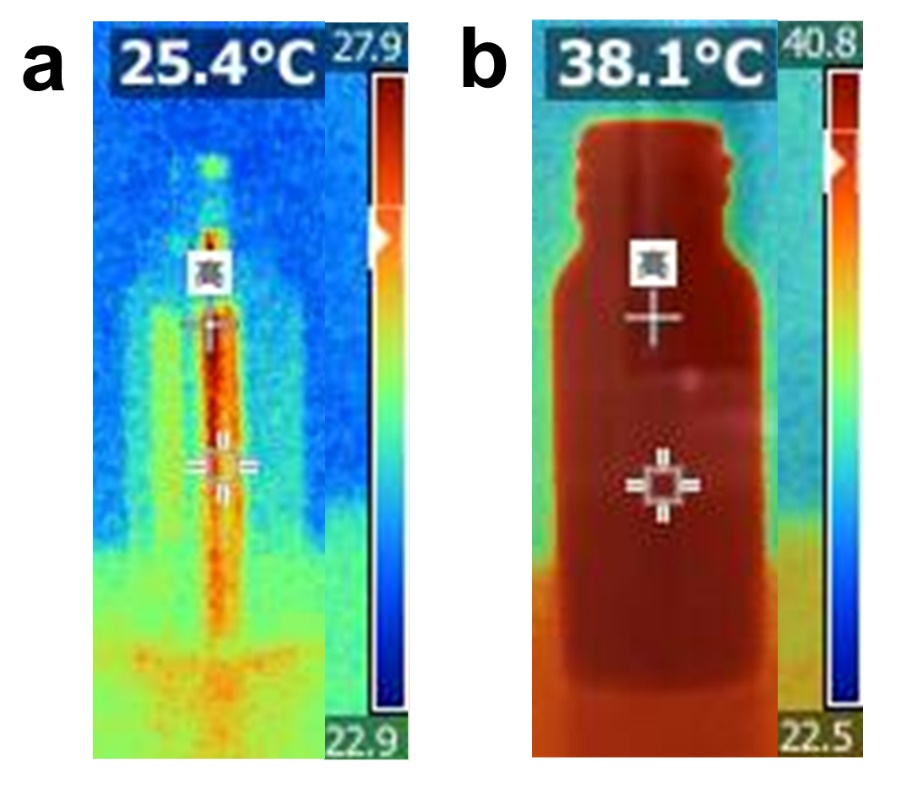


**Figure S5:** Infrared imager photograph of H_2_O (a) and CNTs/H_2_O (b) with 4 min NIR light irradiation ([CNTs]_0_ = 2.0 g L ^−1^ and [distance]_light-bottle_ = 3 cm).


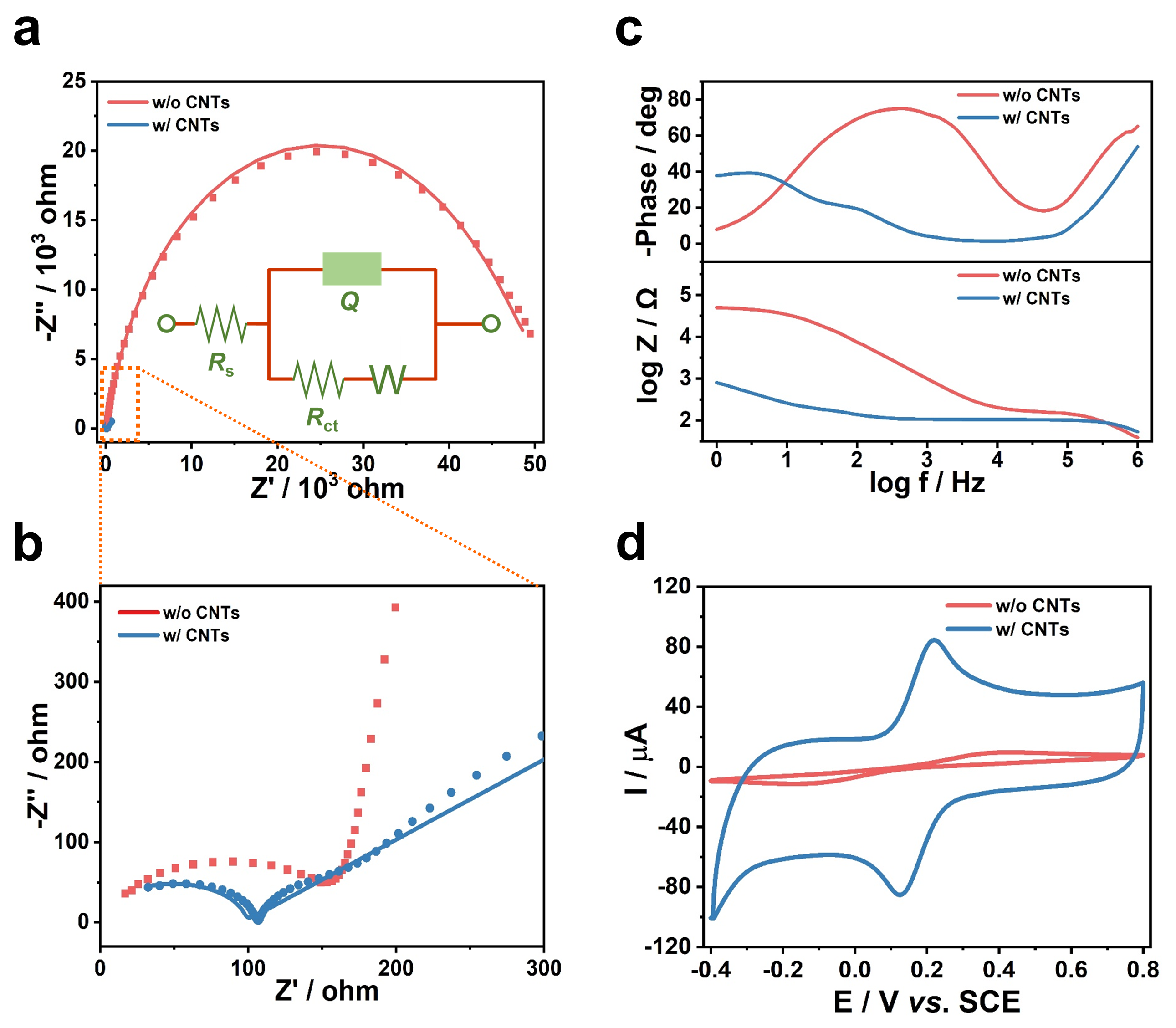


**Figure S6:** Electrochemical characterizations of Au-FOS w/ and w/o coated CNTs. (a) Nyquist plots and Equivalent circuit by simulating EIS. (b) Enlarged version of Fig. S6a. (c) Bode plots, (d) CVs. Supporting electrolyte is 2.5 mM K_3_[Fe(CN)_6_]/K_4_[Fe(CN)_6_] solution containing 0.1 M KCl.

Electrochemical impedance spectroscopy is used to probe the electron transport on the electrodes. The Nyquist plots of different electrodes of Au-FOS w/ and w/o coated CNTs were displayed in Figure S6a-b. The electron transmission capacity of the electrode is reflected by the size of high-frequency arc, in which a smaller arc radius reflects a higher electron transmission capability, indicating the CNTs/Au-FOS with the higher electron transmission capability. The equivalent circuit diagram is used to simulate the EIS data (the inset in Figure S6a), and *R*_s_, *R*_ct_, *Q* and *Z*_w_ represent the resistance of the solution, the charge transfer resistance value between the electrode and solution, the electrode double-layer capacitance and Warburg impedance, respectively. The simulated *R*_ct_ values are shown in Table S2.

Meanwhile, the Bode plot is also a way to compare the charge transfer capability (Figure S6c). For the Bode-magnitude plot, Log |Z| increased with the increasing of the impedance value, suggesting that the CNTs/Au-FOS displays the higher electron transport capability than Au-FOS. The electrochemical behaviors of different electrodes were also evaluated by CVs in Figure S6d. A pair of large reversible redox peaks was observed on CNTs/Au-FOS, compared with Au-FOS electrode, verifying the well electrochemical behavior of as-prepared CNTs/Au-FOS.


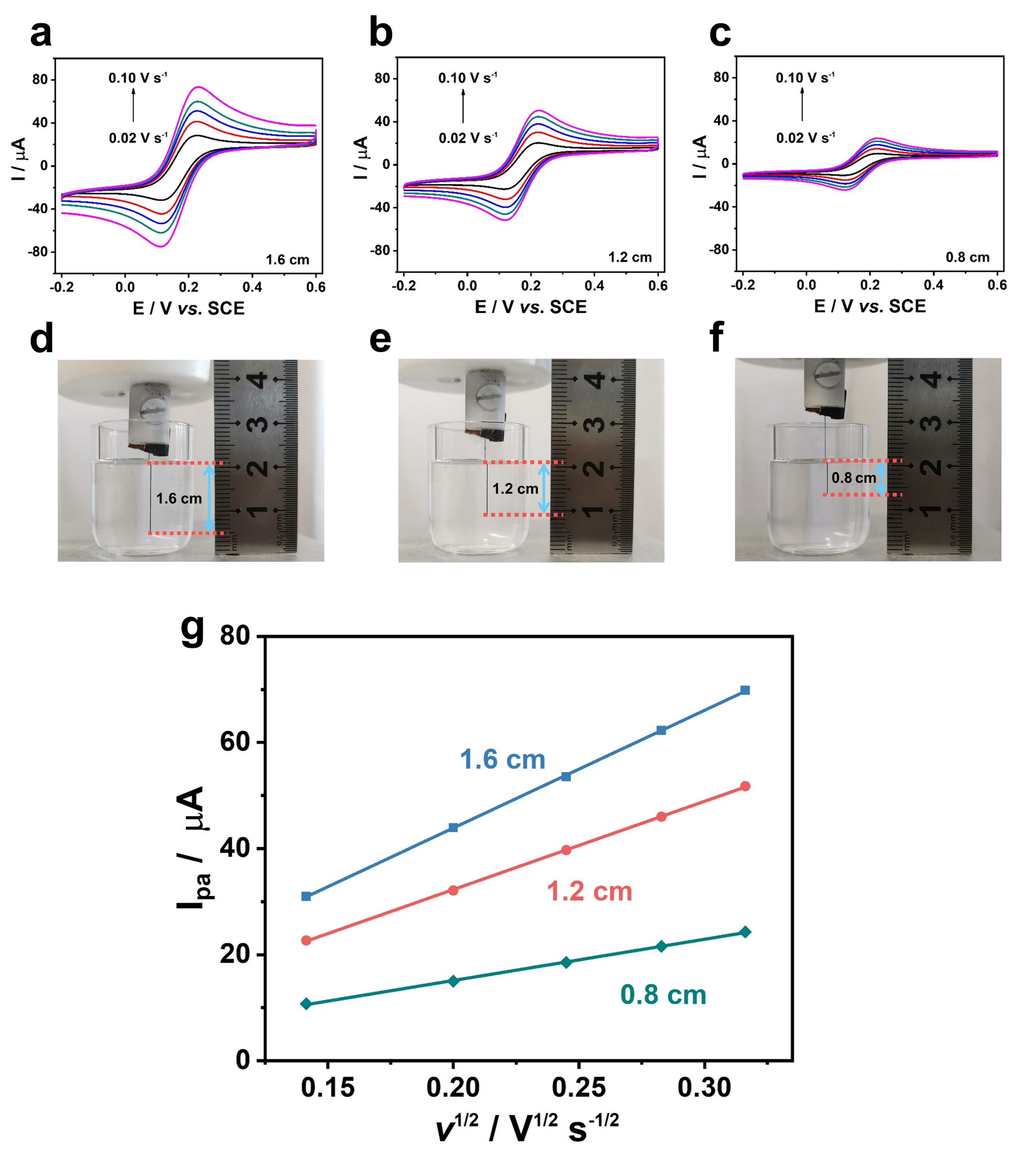


**Figure S7:** Electrochemically active surface area of length of CNTs/Au-FOS submerged in electrolyte. a-f, CV responses and photographs of different length at different scan rate. 1.6 cm (a, d), 1.2 cm (b, e) and 0.8 cm (c, f). g, The relationships of peak current (*I*_pa_) *vs*. *v*^1/2^ (V^1/2^ s^-1/2^). Supporting electrolyte is 2.5 mM K_3_[Fe(CN)_6_]/K_4_[Fe(CN)_6_] solution containing 0.1 M KCl.

Figure S7a-f display that the current value of the oxidation peak (*I*_pa_) of length of CNTs/Au-FOS submerged in electrolyte increased with the increasing of scan rate from 0.02 to 0.10 V s^-1^. The linear relationships between current and square root of scan rate are shown in Figure S7g. The linear regression equations are summarized in Table S3.

Electrochemically active surface area (ECSA) is a crucial factor to evaluate surface reaction of electrode. The Randles-Sevcik equation is used to explore the ECSA of different length. The Randles-Sevcik equation is listed as follows:

*I*_p_ = (2.69×10^5^) *n*^3/2^ *A D*^1/2^ *C v* ^1/2^ (1)

where *n* is the number of electrons participating in the reaction (n=1), *v* is the scan rate (V s^-1^), *A* is ECSA of the electrode (cm^2^), *D* is the diffusion coefficient of K_3_[Fe(CN_6_)] (6.67 × 10^− 6^ cm^2^ s^-1^), *C* is the concentration of the molecule in the solution (mol/cm^3^) and *I*_p_ is the peak current (A). The ECSA is proportional to the value *I*_p_ / *v*^1/2^, which is the slope (also called Randles’ slope) in Figure S7g, and the ECSA of three lengths (1.6 cm, 1.2 cm and 0.8 cm) were calculated as 0.131, 0.0954 and 0.0454 cm^2^, respectively, showing that the deeper the sensor submerged in electrolyte with the larger of ECSA, because of the larger electrochemical reaction area of CNTs. Considering the actual operability of the experiment, a depth of 1.2 cm submerged into the electrolyte for subsequent experiments.


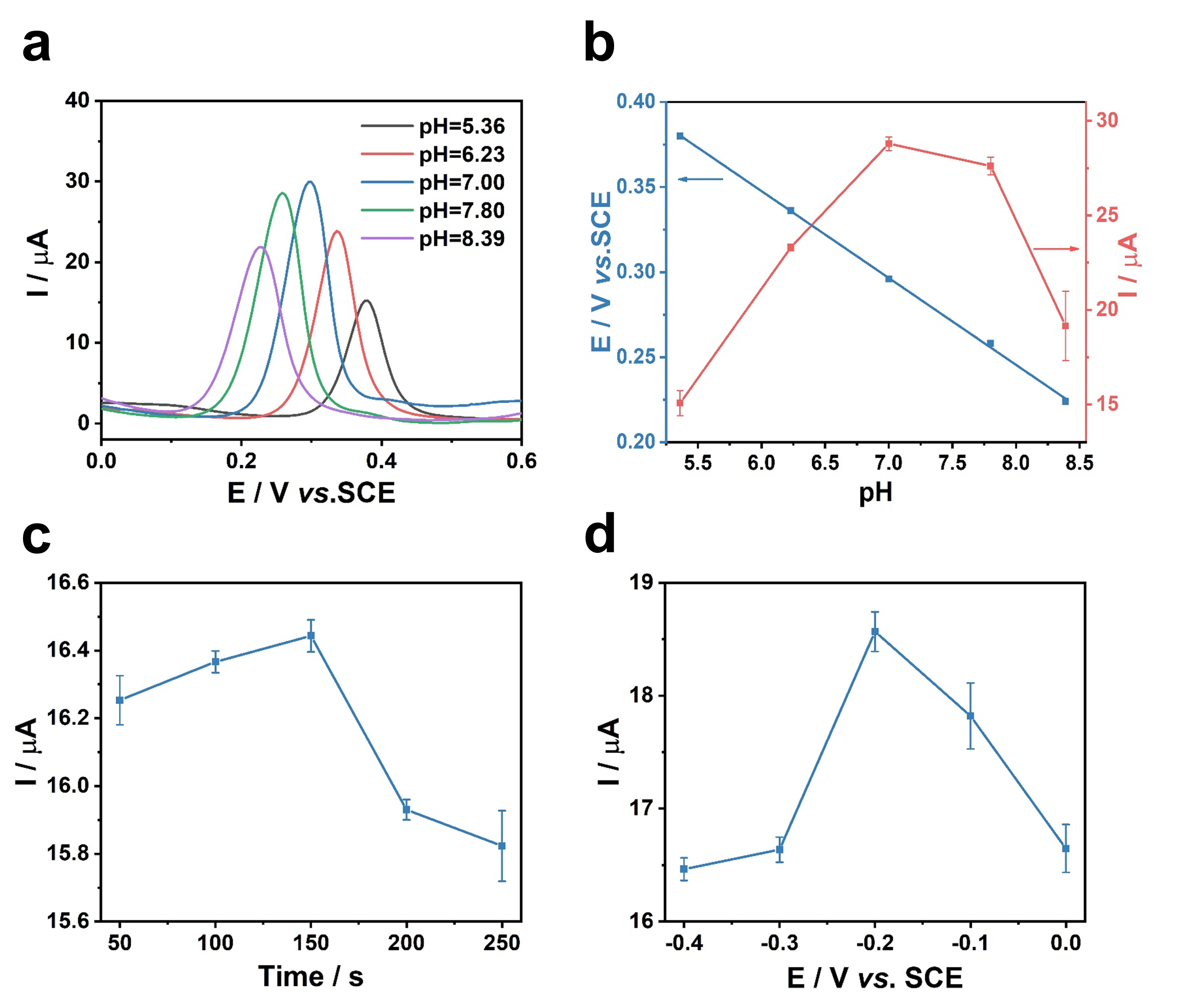


**Figure S8:** Optimization of pH, accumulation potential and accumulation time for CNTs/Au-FOS. a, DPVs behaviors for 20 μM APAP in different pH phosphate buffer. b, Effects of pH value for oxidation current and potential. c, Accumulation time for oxidation current of 20 μM APAP in 0.1 M phosphate buffer (pH = 7.0). d, Accumulation potential for oxidation current of 20 μM APAP in 0.1 M phosphate buffer (pH = 7.0).

Electro-detection conditions including pH, accumulation potential and accumulation time were optimized as follow. It can be seen that the oxidation potential of APAP gradually decreased with the increasing of pH value (Figure S8a, b). The linear relationship between potential and pH can be fitted as *E*_pa_ (V) = (-0.0512 ± 6.826 × 10^-4^) pH + (0.6540 ± 0.0048), R^2^=99.9%. The slope of 51.2 mV/pH was consistent with the theoretical value of 57.6 mV/pH. This result indicates that the transfer process of the same number of electrons and protons can be observed when APAP was oxidized on the sensor surface^1^. Figure S8b shows that CNTs/Au-FOS has the excellent electrochemical properties for APAP detection at pH=7.0. Furthermore, Figure S8c, d display that the highest peak current can be observed at accumulation time of 150 s and -0.2 V for the optimized time and potential.


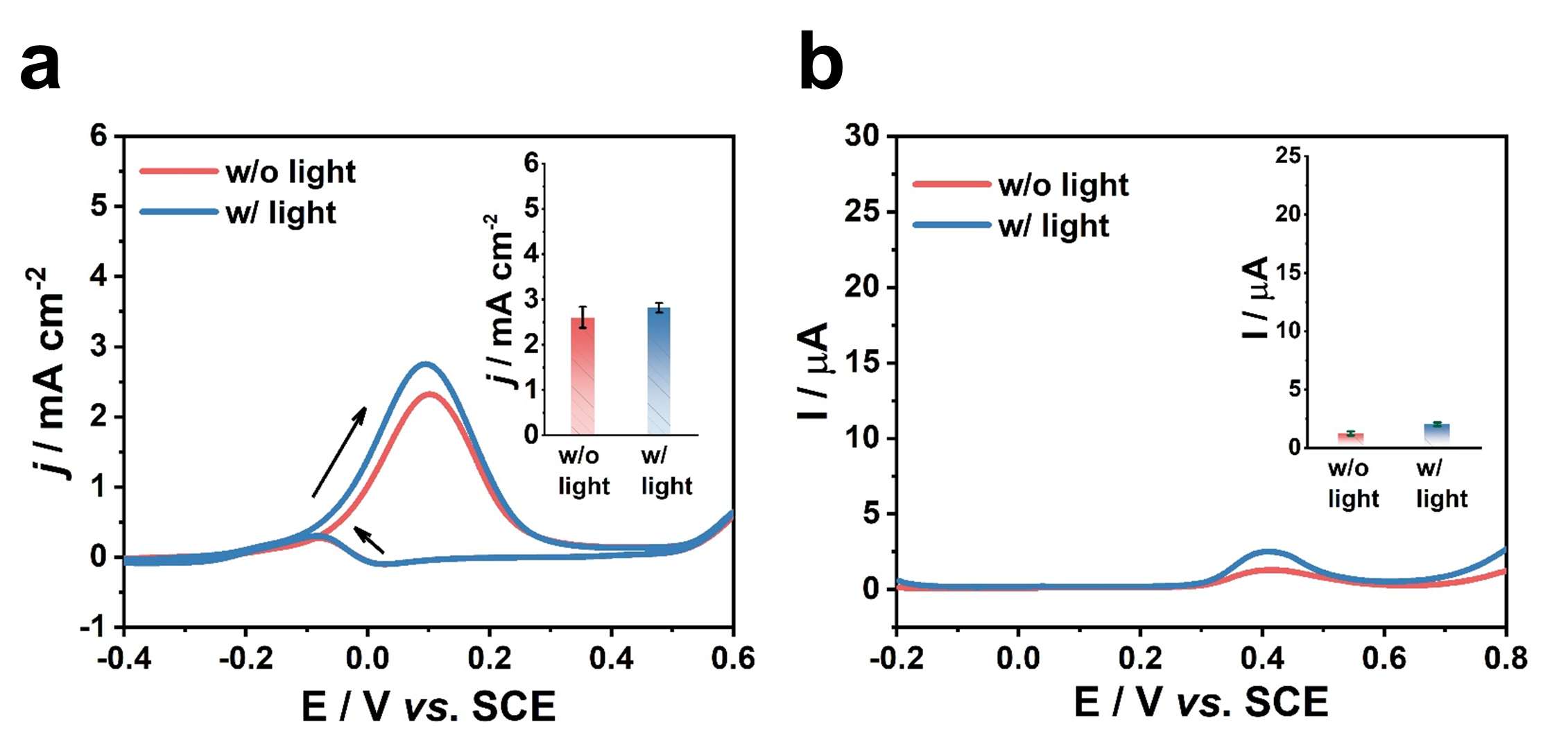


**Figure S9:** Electrochemical reactions of two model reactions on Au-FOS w/ and w/o light. a, CV behaviors in 1 M ethanol and 1 M KOH solution. b, DPV behaviors of 20 µM APAP in 0.1 M phosphate buffer (pH = 7.0). The insets show the corresponding histogram.


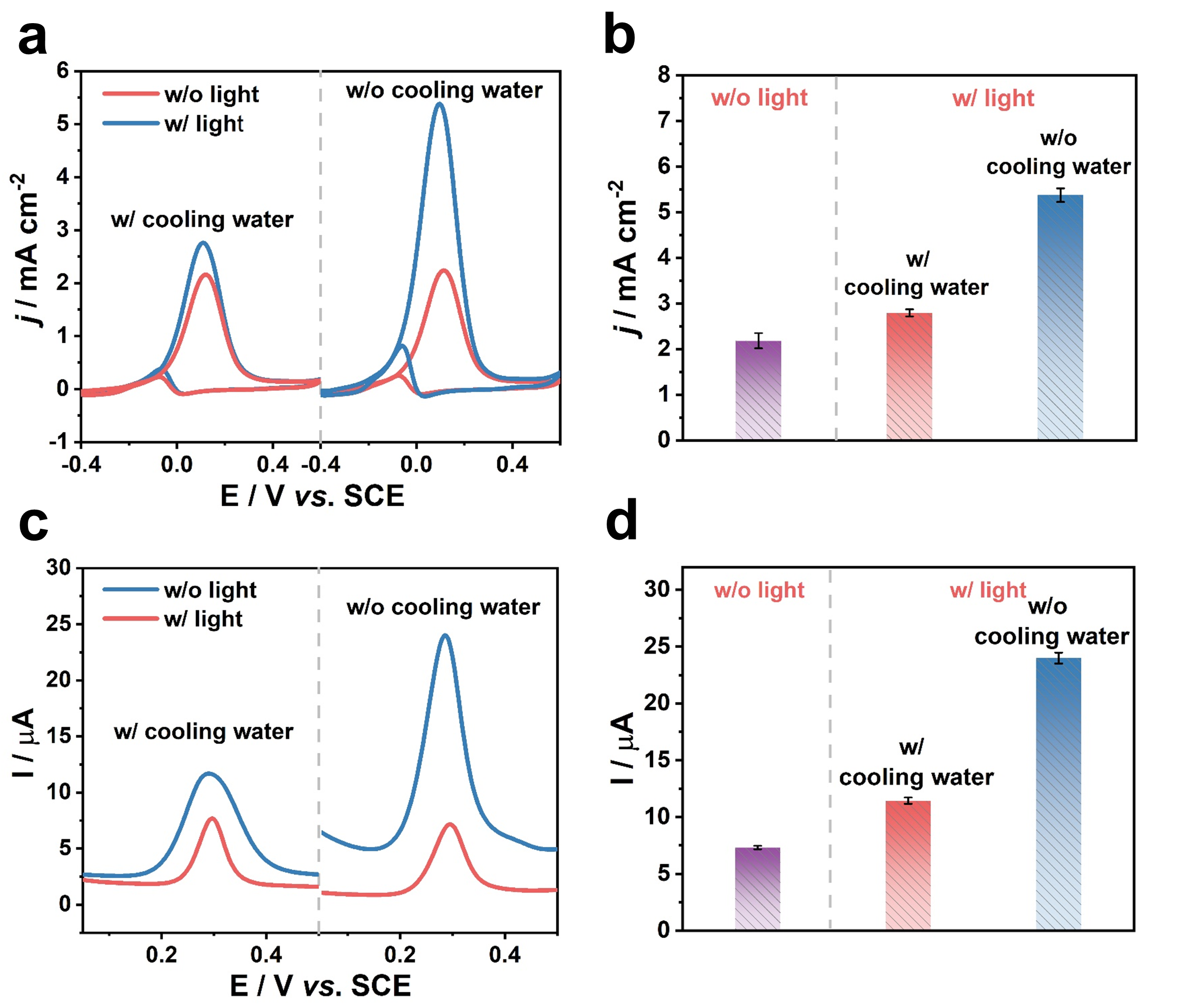


**Figure S10:** Electrochemical reactions of two model reactions on CNTs/Au-FOS w/ and w/o cooling water. a and b, CV behaviors w/ and w/o light in 1 M ethanol and 1 M KOH solution. c and d, DPV behaviors of 20 µM APAP w/ and w/o light in 0.1 M phosphate buffer (pH = 7.0).


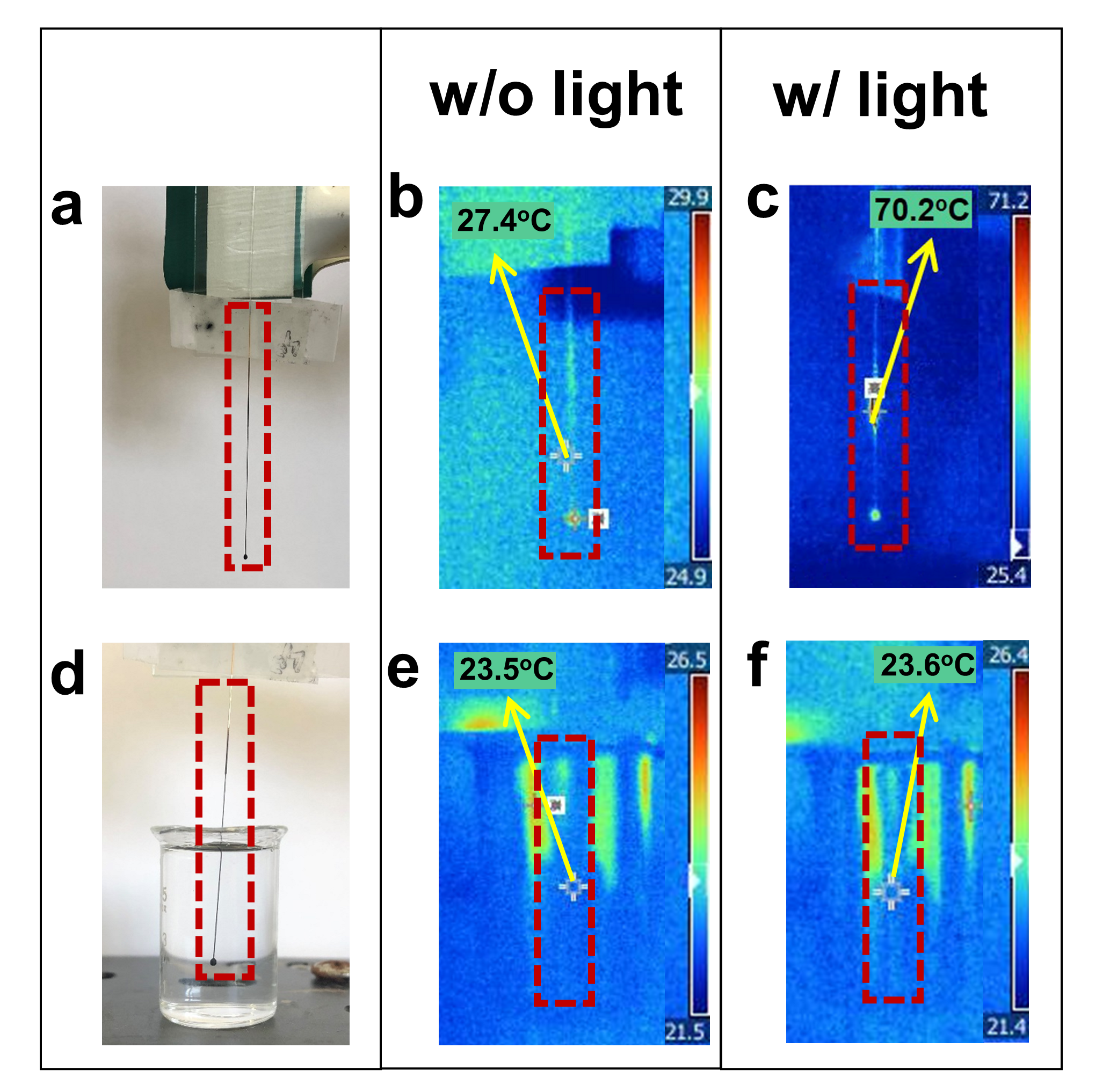


**Figure S11:** Infrared imager photos of CNTs/Au-FOS under different environments with 1 min NIR light irradiation. a and d, Photo of CNTs coated Au-OF in ambient air (a) and water (d) condition; b and c, Infrared imaging photos w/ and w/o NIR light irradiation in ambient air; e and f, Infrared imaging photos w/ and w/o NIR light irradiation in water.

**
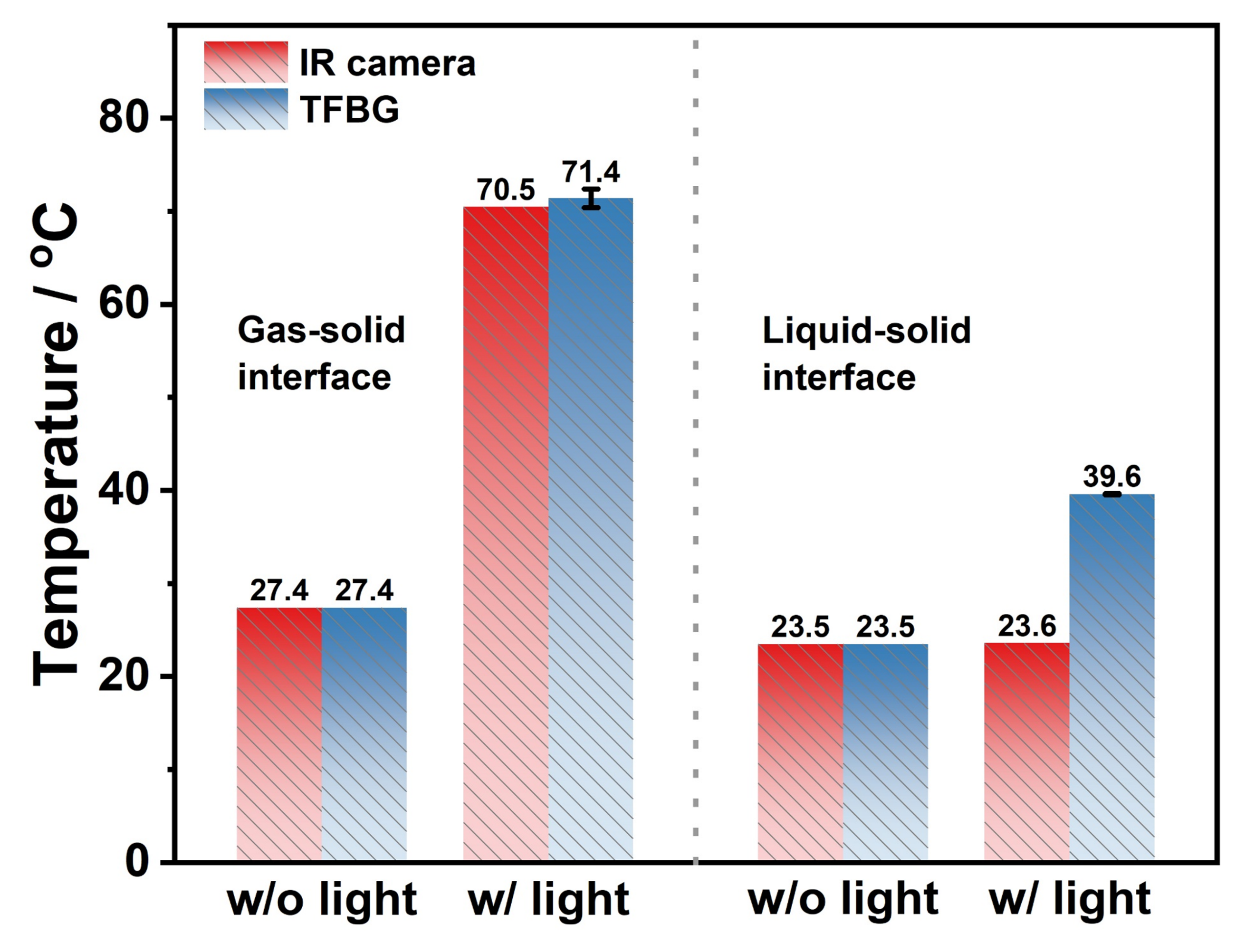
**

**Figure S12:** The comparison of HC mode and infrared imager monitoring the temperatures of CNTs/Au-FOS at the gas-solid interface or liquid-solid interface with 1 min NIR light irradiation.


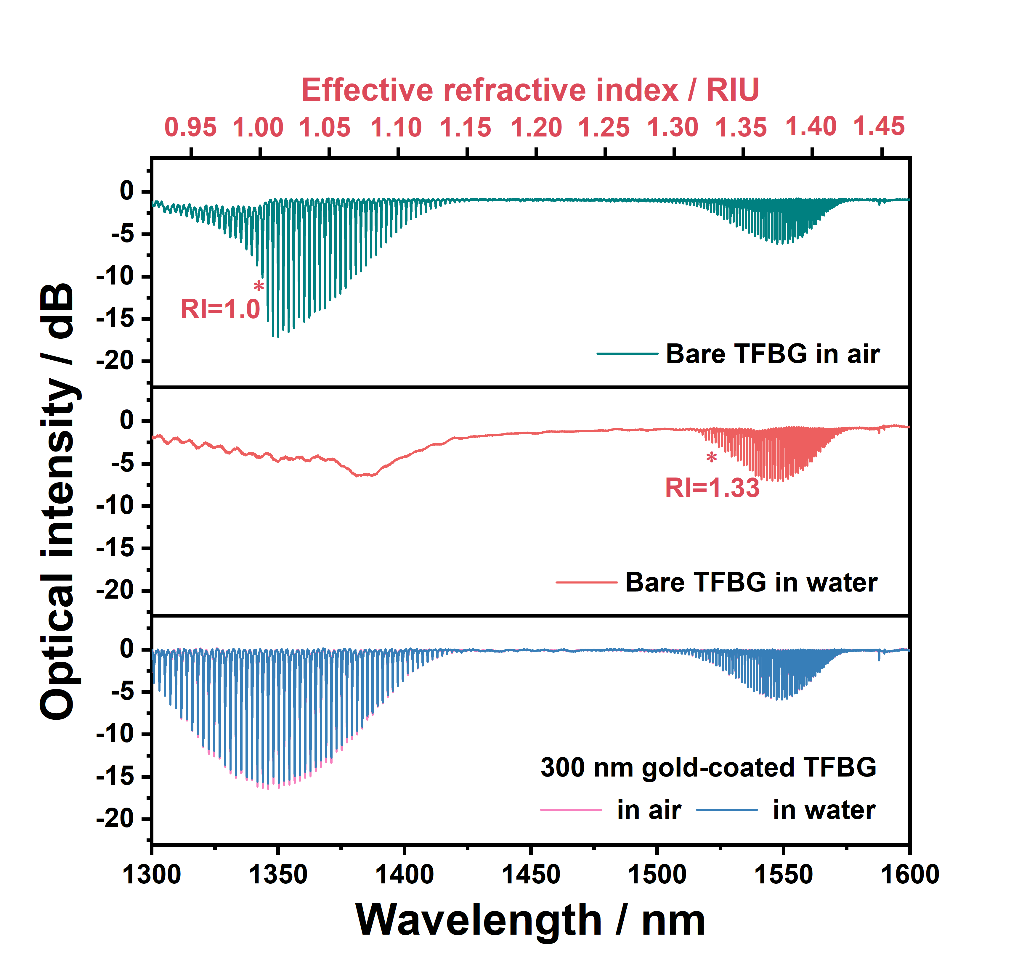


**Figure S13:** Reflection spectrum of the bare TFBG and 300 nm gold-coated TFBG in air and water.


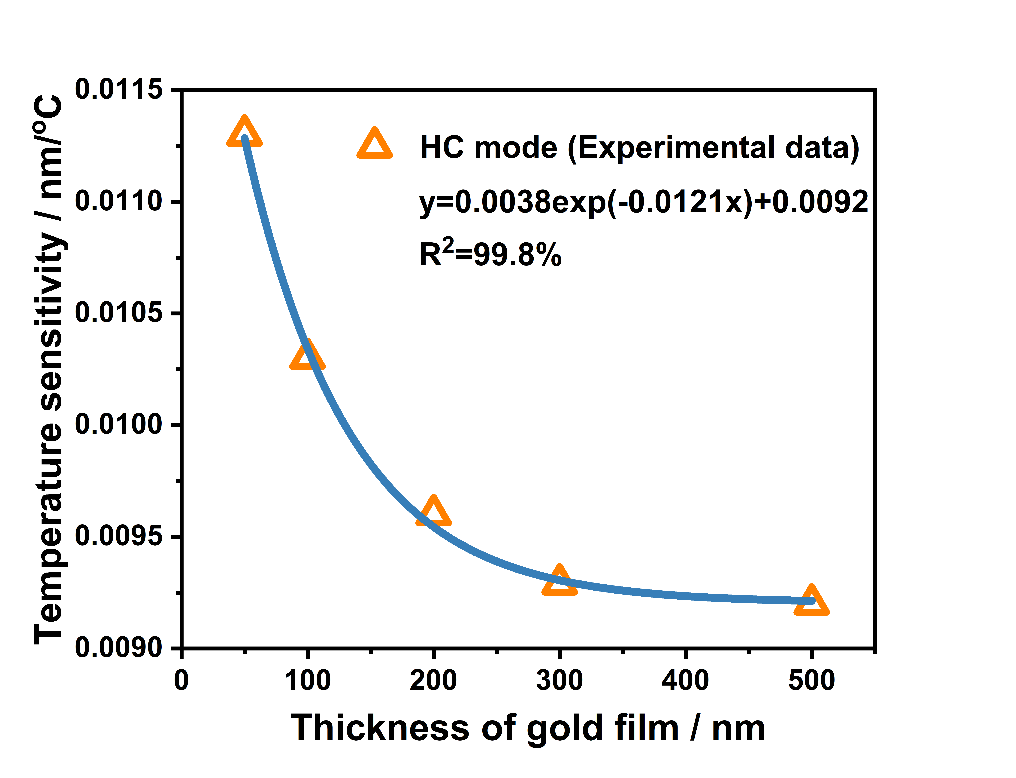


**Figure S14:** Temperature sensitivity of TFBG with the CNT film and different thickness gold films.


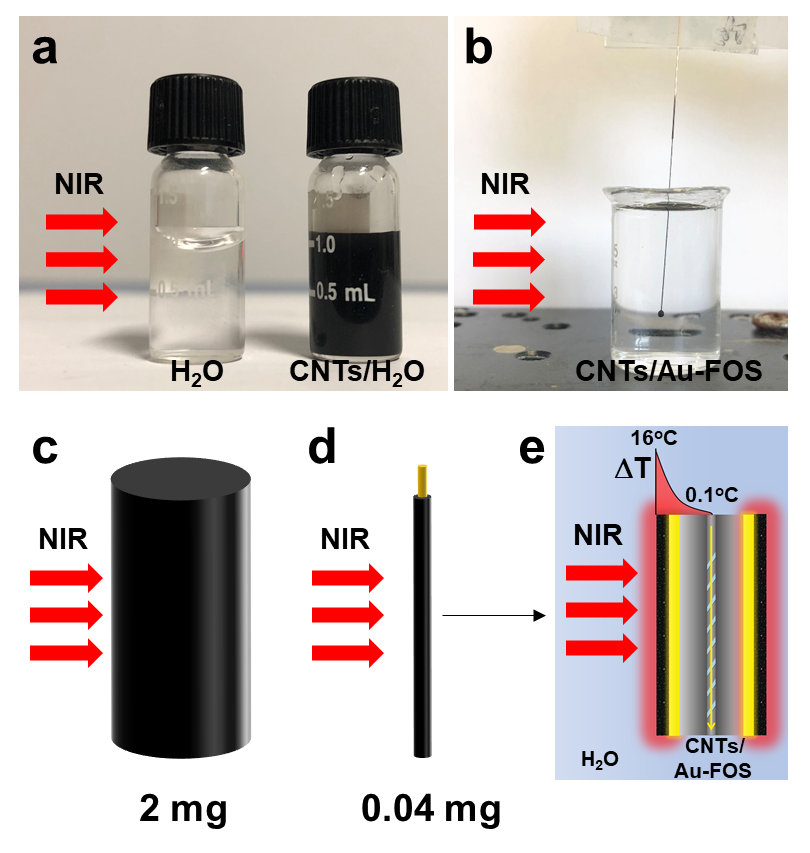


**Figure S15:** Photographs of H_2_O, CNTs/H_2_O (a) and CNTs/Au-FOS (b); Diagram (c and d) of CNTs/H_2_O in a and b; Temperature distribution (e) of CNTs/Au-FOS under NIR illumination in H_2_O.


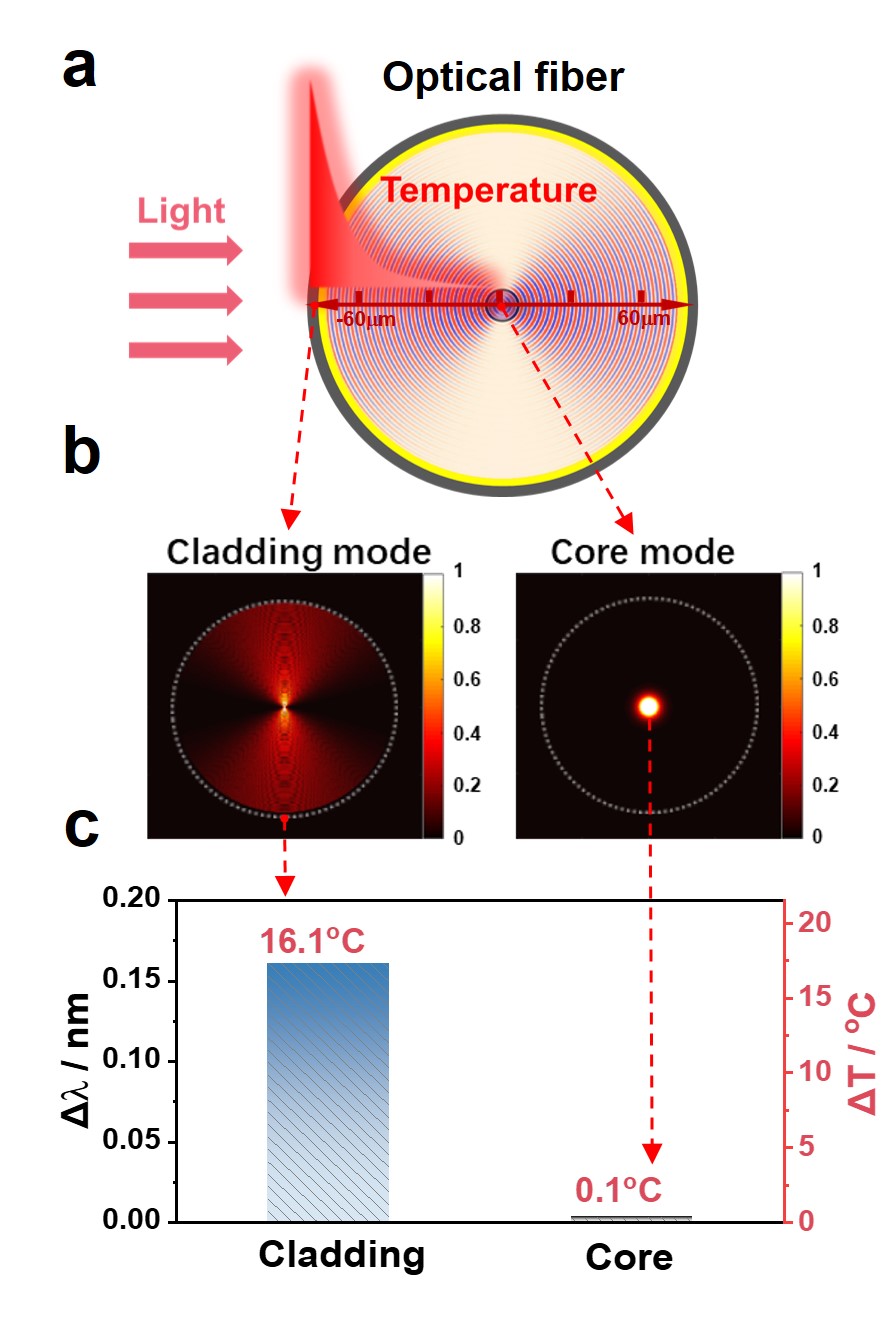


**Figure S16:** The numerical simulation of mode field distributions in the fiber cross section (excited by a TFBG) for light-to-heat measurements and approximate radial temperature distribution (a). The numerical simulation of guided mode field intensity distributions for cladding mode and core mode (b). Temperature increases at the fiber surface determined from the wavelength shift of the HC (99) and core mode following 1 min of light irradiation (c).

**
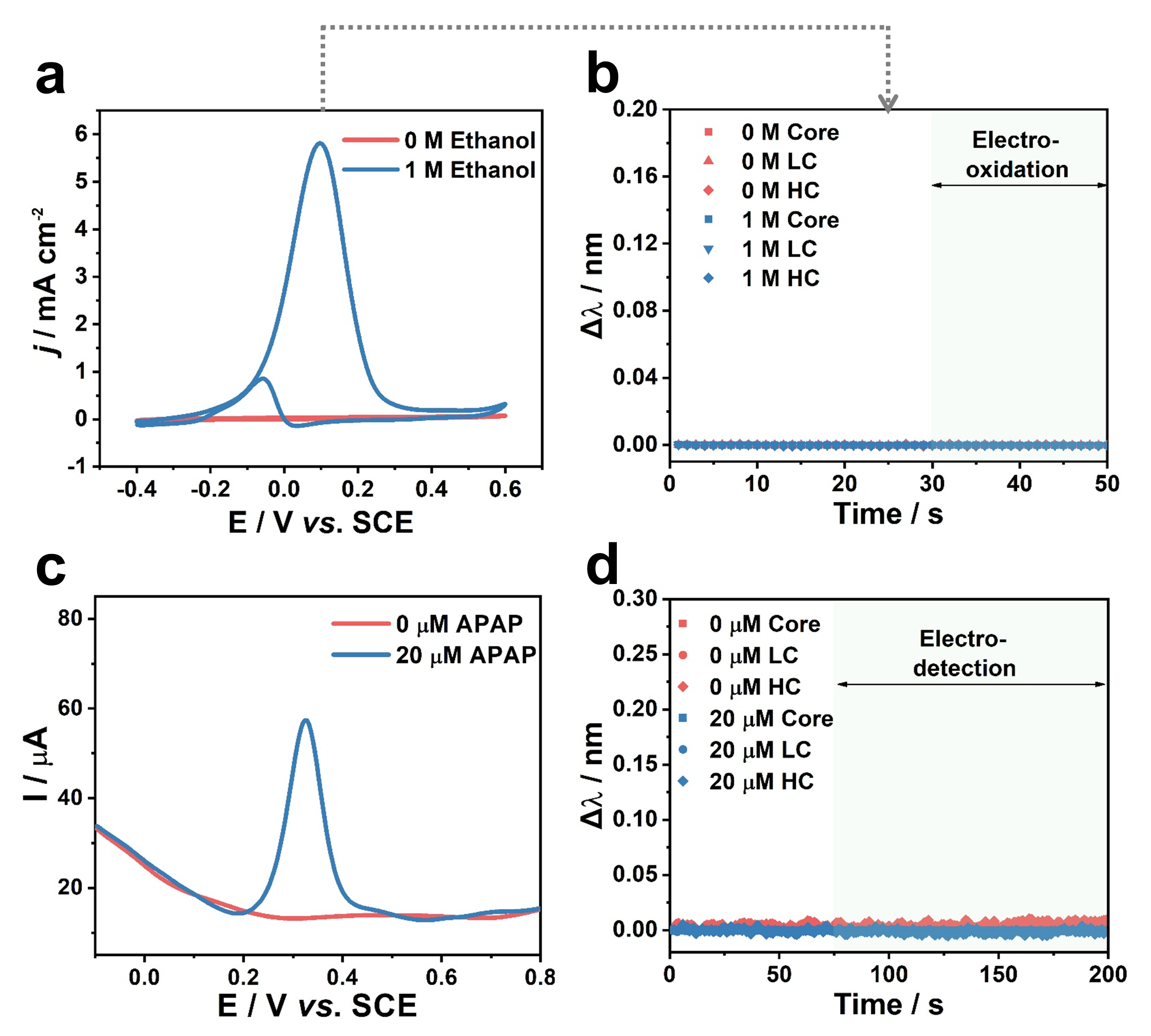
**

**Figure S17:** The changes of refractive index of CNTs/Au-FOS during the electrocatalytic reactions. a, CV behaviors on sensor in 1 M ethanol and 1 M KOH solution. b, Corresponding spectral change process of photo-electrocatalytic ethanol oxidation. c, DPV behaviors of 0 and 20 µM APAP on sensor in 0.1 M phosphate buffer (pH = 7.0). d, Corresponding spectral change process of photo-electrochemical detection of APAP.


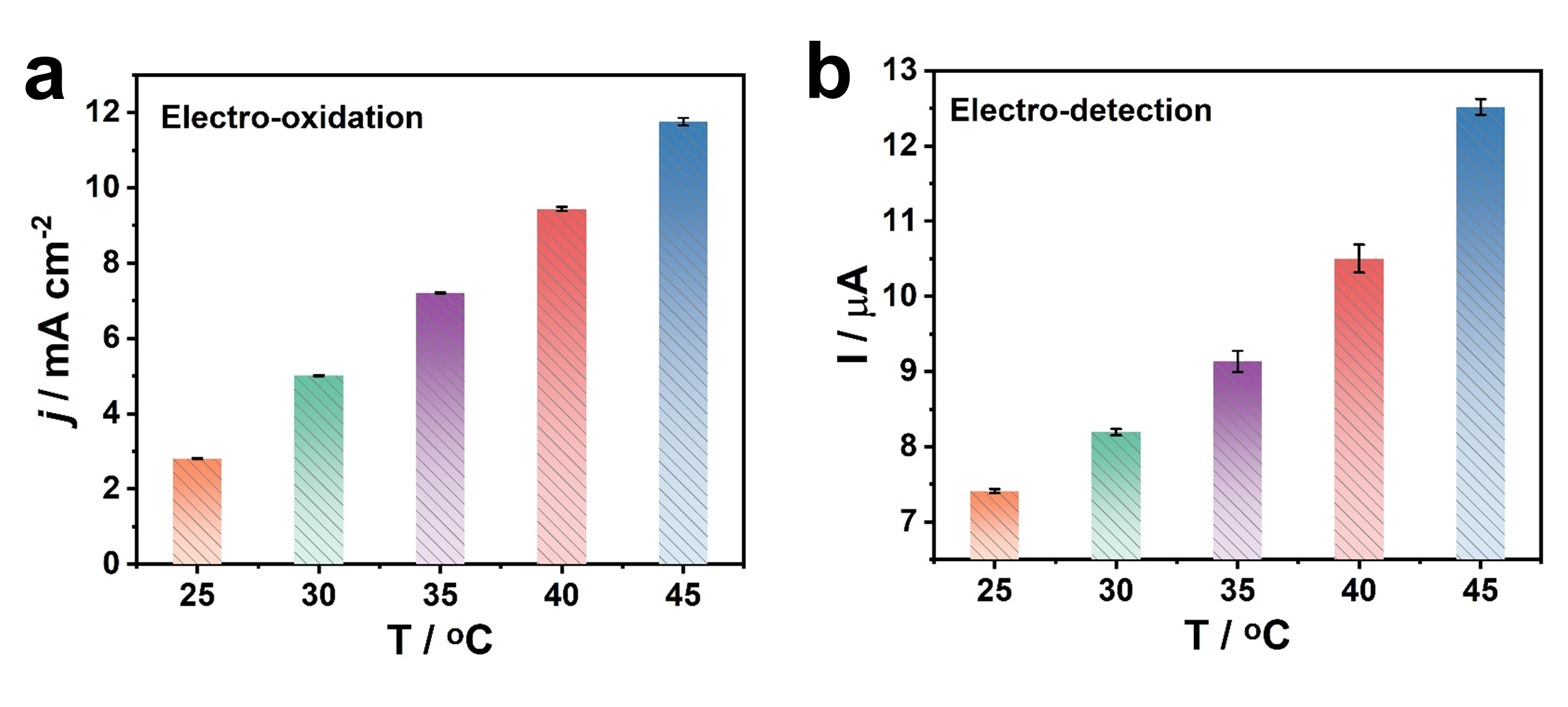


**Figure S18:** Influence of temperature on electrocatalytic reactions of CNTs/Au-FOS by water bath controlled of electro-oxidation for ethanol (a) and electro-detection for APAP (b). Error bars result from the standard deviation from three measurements.


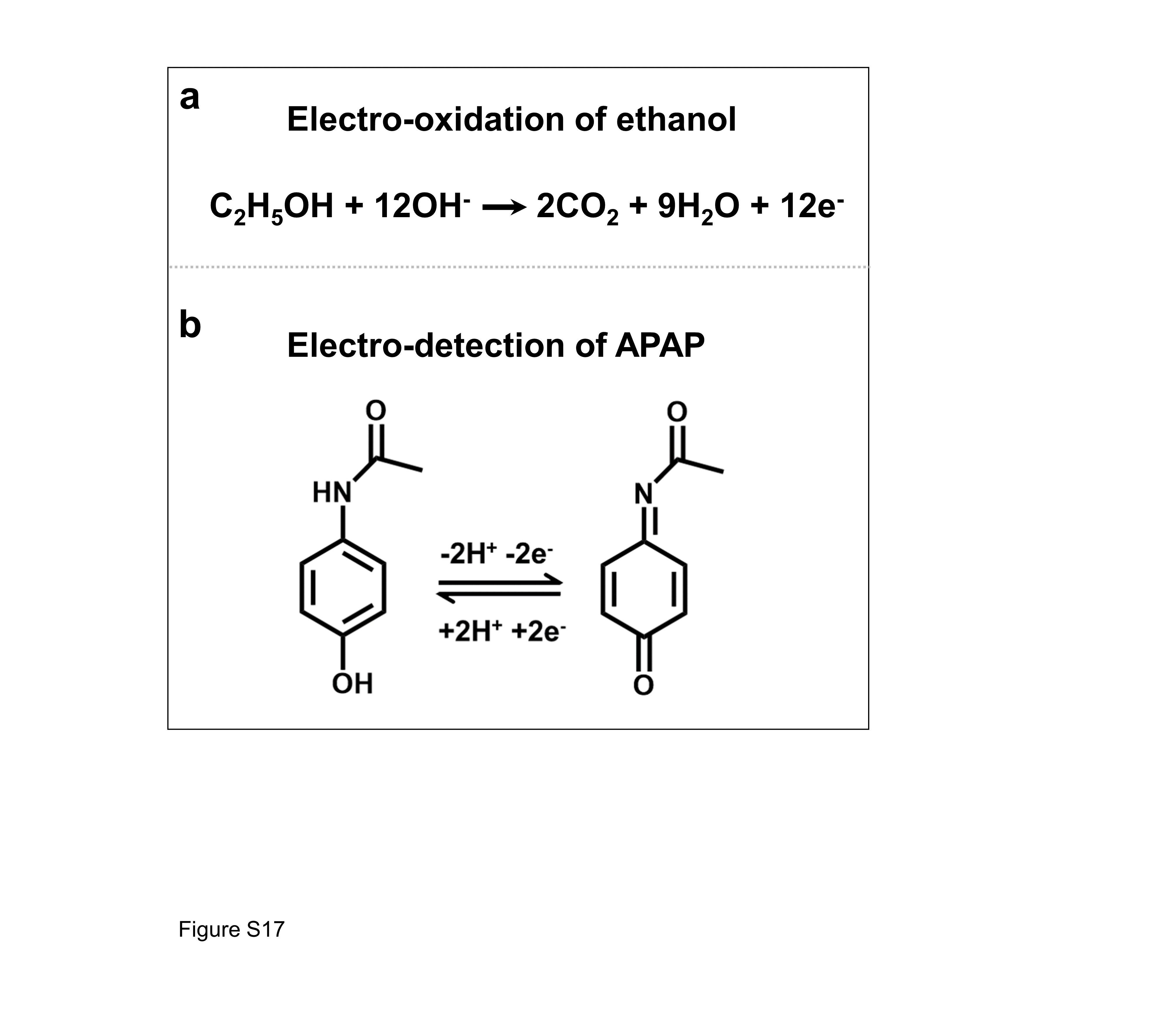


**Figure S19:** The reactions of two electrocatalytic processes of ethanol electro-oxidation (a) and APAP electrochemical detection (b).


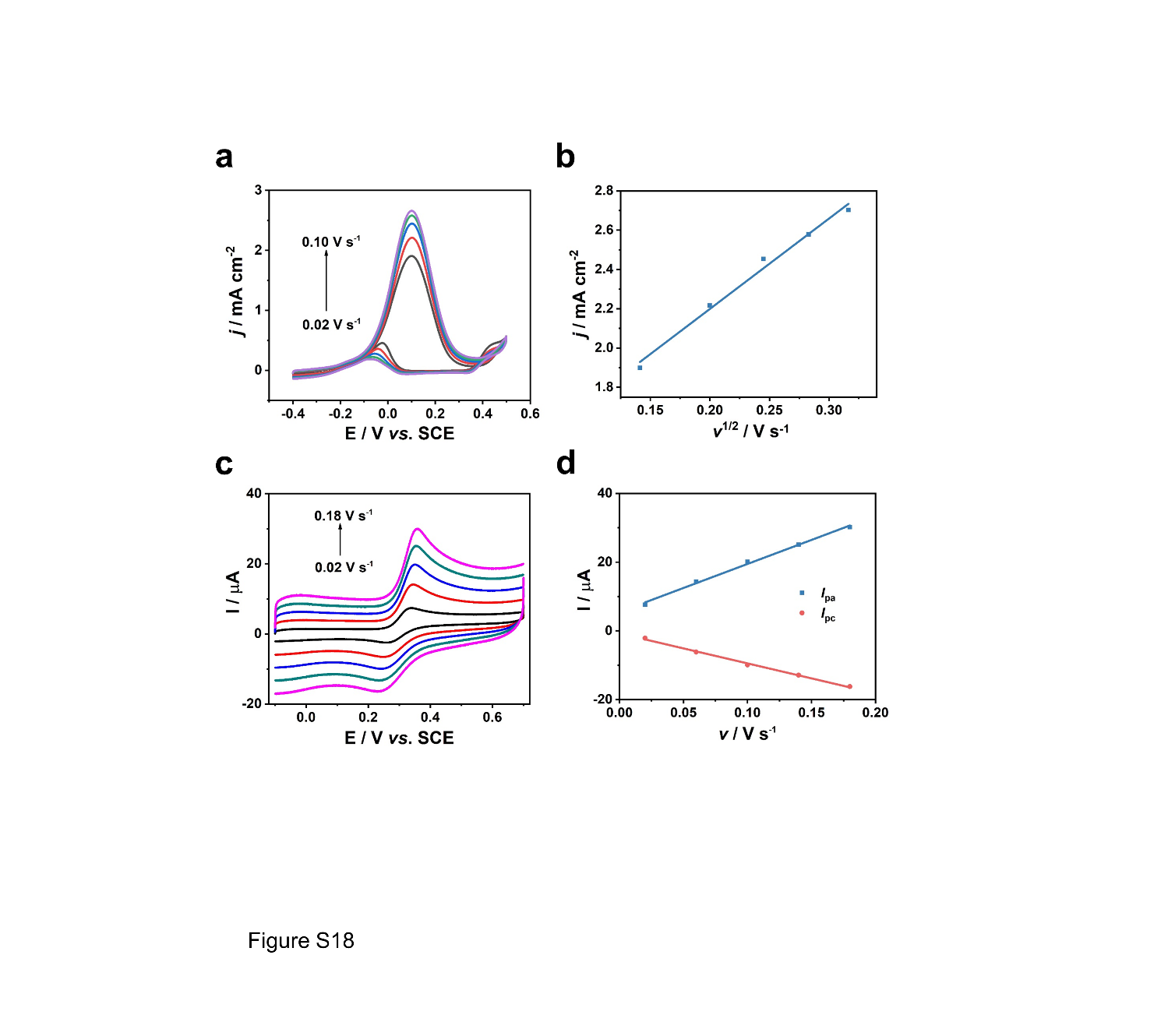


**Figure S20:** Effect of scan rates for two electrocatalytic processes on CNTs/Au-FOS. a, CV responses with different scan rate for 1 M ethanol in 1 M KOH solution. b, The corresponding relationships of *j* *vs*. *v*^1/2^ (V^1/2^ s^-1/2^). c, CV responses of 20 µM APAP with different scan rate. d, The relationships of peak currents (*I*_pa_ and *I*_pc_) *vs*. *v* (V s^-1^).

As presented in Figure S20a and c, the ethanol oxidation shows an irreversible process^2^, and the electrochemical detection of APAP occur a quasi-reversible process^3^. The corresponding linear relationships are displayed in Figure S20b and d, and the regression equations can be expressed as follows:

*j* = (4.605 ± 0.281) *v*^1/2^ + (1.278 ± 0.069), R^2^=98.8% (2)

*I*_pa_ = (114.1 ± 2.1) *v* + (8.57 ± 0.73), R^2^=99.6% (3)

*I*_pc_ = (-71.5 ± 1.1) *v* - (2.49 ± 0.39), R^2^=99.7% (4)

The peak currents for ethanol oxidation are proportional to square root of scan rate, showing that the electro-oxidation of ethanol is a diffusion controlled process^4, 5^. The peak currents for APAP detection are directly proportional to scan rate, indicating that the electrochemical reaction of APAP at the prepared sensor is a surface-controlled process^6, 7^.


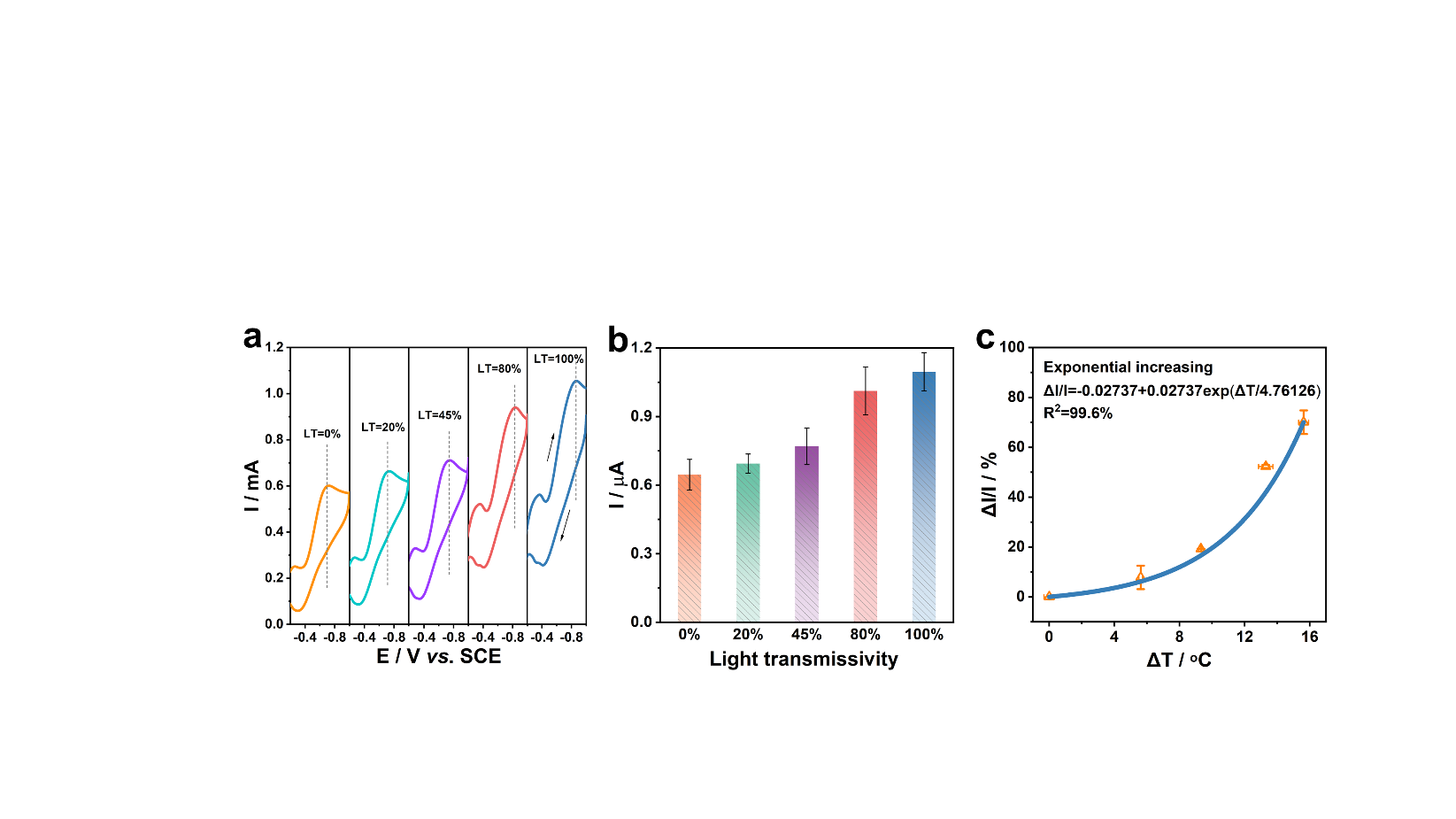


**Figure S21:** a, CV changes of 2 mM H_2_O_2_ in 0.1 M phosphate buffer (pH = 7.0) with increasing LT. b, The corresponding histograms of electrochemical signals *vs*. power level for H_2_O_2_ detection. c, Relationship between electrocatalytic activity of H_2_O_2_ detection and the catalyst surface temperature. Error bars result from the standard deviation from five measurements.


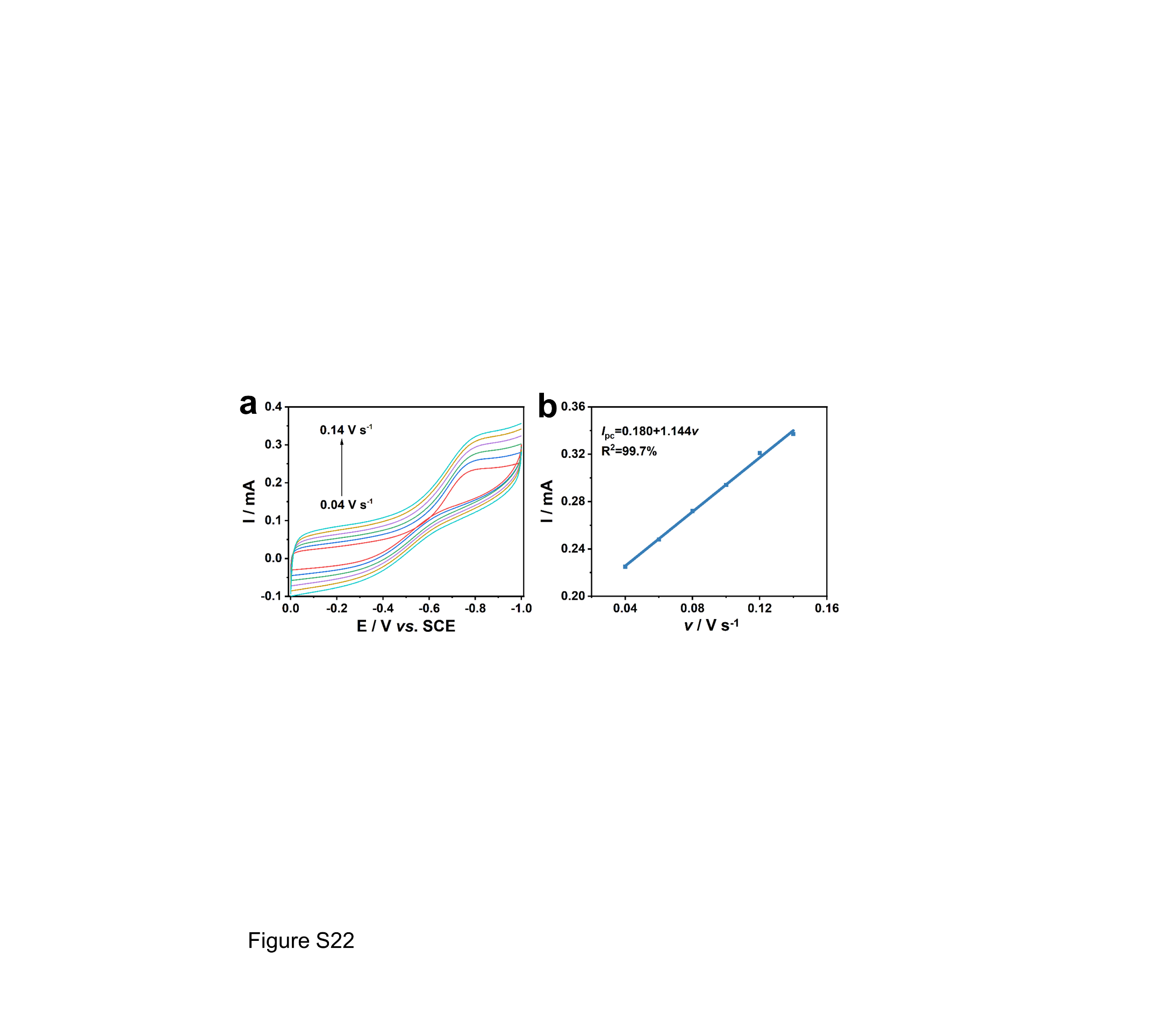


**Figure S22:** Effect of scan rates for H_2_O_2_ detection on CNTs/Au-FOS. a, CV responses with different scan rate for 1 mM H_2_O_2_ in 0.1 M phosphate buffer (pH = 7.0). b, The relationships of peak currents (*I*_pc_) *vs*. *v* (V s^-1^).

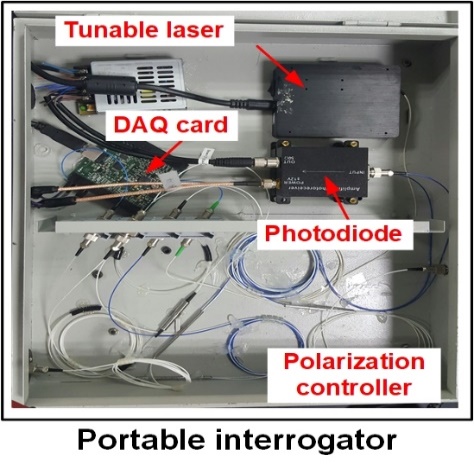


**Figure S23:**  High speed SPR interrogation scheme (up), spectral response (bottom, left) and its prototype (bottom, right) in which the acquisition time can reach to 5 ns.

**Figure S24:**  The experimental transmission spectra in air as a function of tilted angle. The tilt of the grating fringes was obtained by rotating the fiber and phase mask around an axis perpendicular to the plane containing both the fiber axis and the laser beam at an angle of such that the external tilt angle is ranging from 5o to 40o. When rising the tilt angle of grating, higher order cladding mode (smaller effective mode refractive index, located at shorter wavelength) will be excited. Meanwhile, at the 37-degree TFBG there is a clear cut-off where the cladding mode resonances decrease sharply in amplitude, indicating loss of total internal reflection at the point where the cladding mode effective index becomes equal to air.

**Supplementary Tables**

**Table S1.** The fit parameters of two catalytic processes in Figure 6e and Figure 6f.

| Catalytic process | Equations |
| --- | --- |
| Electro-oxidation | $\Delta j/j=0.08934 \Delta T-0.00366$ |
| Electro-detection | $\Delta I/I=0.2724 exp\left( \left( \Delta T+0.38589 \right)/7.56095 \right)- 0.28216$ |

**Table S2.** *R*_ct_ values for different sensors.

| Sensor | *R*_s_ (Ω) | *R*_ct_ (Ω) |
| --- | --- | --- |
| Au-FOS | 140 | 4.86×10^4^ |
| CNTs/Au-FOS | 0.895 | 96.0 |

**Table S3.** The linear relationships between current and scan rate square root and electrochemically active surface area (ECSA).

| Length / cm | Linear equation | ECSA / cm^2^ |
| --- | --- | --- |
| 1.6 | *I*_pa_ (μA) = (228.03 ± 4.48) *v*^1/2^ (V^1/2^ s^-1/2^) _ (1.72 ± 1.19) (R^2^ = 0.998) | 0.1313 |
| 1.2 | *I*_pa_ (μA) = (165.78 ± 0.89) *v*^1/2^ (V^1/2^ s^-1/2^) – (0.86 ± 0.24) (R^2^ = 0.999) | 0.0954 |
| 0.8 | *I*_pa_ (μA) = (78.85 ± 1.13) *v*^1/2^ (V^1/2^ s^-1/2^) – (0.63 ± 0.30) (R^2^ = 0.999) | 0.0454 |

**Supplementary References**

1. Zhang W, Liu S, Zhang Y, Ding X, Jiang B, Zhang Y. An electrochemical sensor based on electro-polymerization of caffeic acid and Zn/Ni-ZIF-8–800 on glassy carbon electrode for the sensitive detection of acetaminophen. *Biosens. Bioelectron.* **131**, 200-206 (2019).

2. Barbosa A, Oliveira V, Van Drunen J, Tremiliosi-Filho G. Ethanol electro-oxidation reaction using a polycrystalline nickel electrode in alkaline media: temperature influence and reaction mechanism. *J. Electroanal. Chem.* **746**, 31-38 (2015).

3. Alam AU*,* Qin Y, Catalano M, Wang L, Kim MJ, Howlader MMR, Hu N-X, Deen MJ. Tailoring MWCNTs and β-cyclodextrin for sensitive detection of acetaminophen and estrogen. *ACS Appl. Mater. Inter.* **10**, 21411-21427 (2018).

4. Wang M, Xiao F-N, Wang K, Wang F-B, Xia X-H. Electric field driven protonation/deprotonation of 3, 4, 9, 10-perylene tetracarboxylic acid immobilized on graphene sheets via π–π stacking. *J. Electroanal. Chem.* **688**, 304-307 (2013).

5. Zhai C*, et al.* Construction of Pt/graphitic C_3_N_4_/MoS_2_ heterostructures on photo-enhanced electrocatalytic oxidation of small organic molecules. *Appl. Catal. B: Environ.* **243**, 283-293 (2019).

6. Lu T-L, Tsai Y-C. Sensitive electrochemical determination of acetaminophen in pharmaceutical formulations at multiwalled carbon nanotube-alumina-coated silica nanocomposite modified electrode. *Sensor. Actuat. B: Chem.* **153**, 439-444 (2011).

7. Xu Z*, et al.* Density Functional Theory-Assisted Electrochemical Assay Manipulated by a Donor–Acceptor Structure toward Pharmaceutical Diagnostic. *Anal. Chem.* **92**, 15297-15305 (2020).
